# Image adaptive encryption using EfficientNet B3 feature guided multi scroll chaotic map with modulo controlled pseudo parallel processing

**DOI:** 10.1038/s41598-025-27080-z

**Published:** 2025-12-06

**Authors:** S. Subathra, V. Thanikaiselvan

**Affiliations:** School of Electronics Engineering, Vellore Institute of Technology, Vellore, Tamil Nadu 632014 India

**Keywords:** EfficientNet-B3, Key generation, Image encryption, Bidirectional selective confusion, Chaotic intra/inter pixel diffusion, Dynamic DNA coding, Computer science, Information technology

## Abstract

A new multistage encryption algorithm is proposed by integrating the deep neural network with a new 4D multi-scroll chaotic map to enhance the efficiency and improve the security of image transmission in the open channel. This combined network expands the key space and maintains the secrecy of the key with the multistage encryption algorithm. Initially, the image adaptive key generation process is implemented by the EfficientNet-B3 network to extract the features from the source image, which are then converted into hash values using SHA 256. The hash values are partitioned into four sections, and each section is normalized to give one distinct initial value for the generation of a multi-scroll chaotic sequence. The pseudo-parallel process routes the split source sub-image blocks (128⨯128) of plain text to branch 1 or branch 2, decided by the seed value of the chaotic sequence, increasing the high robustness against the differential and statistical attacks. Each branch contains row and column-wise permutations, bidirectional selective shuffling, and chaotic intra/inter-pixel diffusion in varying orders. The key image diffusion and dynamic DNA diffusion to the intermediate cypher image exhibit a strong avalanche effect. The simulation evaluation on the natural data set images demonstrates the large key space of 2 to the power of 674, high key sensitivity, uniform histogram with entropy value attains the critical values of 7.9, high NPCR value of 99.9%, UACI values with 33.46%, almost zero-pixel correlation and strong robustness to the cropping and noise attacks.

## Introduction

Rapid advancements in web-based platforms and digital media frameworks have substantially improved performance and simplified information communication and storage. However, several underlying security issues remain. Images are vital to multimedia data and contain sensitive information, such as private medical records, proprietary Visual Data, and personal information. Various digital photos from several fields on the Internet are sensitive to threats like content leaks and illegal access. Image encryption plays a crucial role in solving this challenge. Since there are more devices than humans and attackers are getting smarter, it is challenging to implement adequate information security measures nowadays. Confidentiality, authenticity, and integrity are some of the security elements that influence the security algorithms used to safeguard the transfer of multimedia files, including text, images, audio, and video. Data exchange and preservation have become essential components of our everyday lives in the modern era of fast technological growth. As a result, scientists have initiated the exploration of new techniques for image encryption. Typical methods include picture encryption schemes that use hyperchaotic encryption^1,2^, deep learning secure methods^3^, wavelet transform encryption^4^, and others. The large data size, redundant pixel distribution, and Inter-pixel correlation of images make traditional encryption technologies, which are helpful for text data, ineffective at protecting the digital photos.

The development of encryption techniques for protecting photographs is of great interest to many researchers. In several cryptosystems, chaotic systems have been suggested as strong encryptors due to their exceptional performance, which includes high degree of ergodicity and sensitivity to the initial parameters^5^. The paper^6^, suggested improved one dimensional chaotic equation for bit level permutation and diffusion. The research work^7^ proposed a new digital Chebyshev chaotic system that combines the digital logic system with digital Chebyshev’s chaotic system results in high information entropy and increases chaotic properties. In paper^8^, a new two-dimensional enhanced cosine-sine-logistic model(2D-ECSLM) is implemented to improve the performance of self-reversible secured image property. This research work^9^ proposed a new 1D-Quadratic Sine (1DQS) chaotic system with parallel permutation and dispersion to improve the computation speed of the algorithm. The main vulnerability to these techniques that can result in the encryption system failure is the incorporation of low-dimensional chaotic systems (i.e., 1D or 2D), which have a small key space, short periodicity, and a high probability of secret key guessing. However, higher-dimensional (3D or more) chaotic systems contain many parameters, a complex structure, a huge key space, and a high sensitivity, which makes it challenging to crack picture cryptosystems^10,11^. The paper^12^ demonstrates a novel 3-Dimensional hyperchaotic system with integer wavelet transform to enhance the robustness of medical image encryption. The 4D large-scale hyperchaotic map (4D-LSHM) is integrated with compressed sensing to increase encryption efficiency, as demonstrated in the research work^13^. Numerous researchers have evaluated encryption methods by combining chaotic systems and DNA encoding, which have more storage size, secure encrypted systems, and Massive Parallelism^14^. Gera U.K. et al.^15^ have implemented a 4D hyperchaotic sequence with DNA arithmetic operation to improve diffusion technique in the encryption algorithm. Conventional methods, such as biometric-based encryption systems^16^, generation of random numbers, and static keys^17^, frequently encounter drawbacks like predictability, lack of flexibility, and susceptibility to known plaintext or brute-force attacks.

Deep learning-based intelligent encryption is becoming increasingly used to encrypt images in different communication platforms. Significant promise exists for enhancing cryptography methods by integrating deep neural network design with chaotic systems. To safeguard privacy while computation is underway, several deep learning-based image encryption techniques have been developed^18^. In the paper^19,20^ the research works proposed chaotic system with neural network to safeguard the visual data by providing robust encryption technique. Complex and High-Reliability Encryption algorithms that are difficult for unauthorized parties to decipher can be created by Artificial Intelligence (AI). However, by increasing computational complexity, AI’s ability to protect images depends on the size and quality of training datasets^21,22^.

Besides the advantages of existing techniques, the literature reveals evident limitations, including small key space results due to the inefficient generation of keys. Multidimensional chaotic systems generate an ample key space, but as computational time increases, some systems lead to efficiency limitations in image encryption security. This proposed work addresses the current issue by integrating a multi-level encryption phase, using a novel image adaptive key generation process with EfficientNet-B3, a New multi-scroll chaotic map, a pseudo-parallel process of the image, three different stages of permutation, modified chaotic intra-pixel diffusion and inter-pixel diffusion, key image diffusion, and dynamic DNA XOR diffusion.

To ensure that each input image generates a unique cryptographic key, we develop a novel image encryption framework that uses EfficientNet-B3 to extract deep features from grayscale images. A subset of these features is then converted into a hash value using SHA256. The 32-byte hash value is divided into 4 segments, each 64 bits long. Each 64-bit segment is then normalised and mapped into four adaptive initial conditions for multi-scroll chaotic systems. Furthermore, we incorporate a multi-scroll chaotic map, which is renowned for its high-dimensional complexity and better key space expansion, to improve diffusion and confusion properties in the encryption process. To enhance the efficiency of the proposed work, modified chaotic inter-pixel diffusion and intra-pixel diffusion are implemented. This flexibility not only adds security but also improves resistance to statistical and differential attacks.

**Significant contributions of this proposed method:**

- EfficientNet-B3 is proposed as an image adaptive key generation technique for the proposed image encryption algorithm.
- The New Multi-Scroll chaotic map is developed to enhance the complexity and unpredictability of key-driven encryption.
- The randomness of the proposed New Multi-Scroll chaotic map, is verified using the NIST SP 800-22 statistical test suite
- A pseudo-parallel encryption structure is introduced to improve efficiency without compromising algorithmic robustness
- This proposed multistage encryption method randomly distributes the pixels using the chaotic points generated, with the innovative Chaotic Intra-Pixel Diffusion (CIPD) and Inter-Pixel Diffusion (CIPD), which improves security.
- The multistage encryption enlarges the adequate key space by combining four image-adaptive initial conditions, five control parameters, and a 256-bit SHA-256 hash, yielding an estimated key space on the order of 10^202 ^≈ 2^674^ which strengthens resistance to brute-force attacks.
- Experimental results confirm superior entropy, sensitivity, and diversity performance compared with conventional key-generation techniques

The structure of this paper is as follows: Section "Preliminaries" introduces the 4D multi-scroll chaotic map and the EfficientNet-B3 based key generation. Section "Proposed algorithm" details the proposed encryption framework and processing stages. Section "Results and performance analysis" reports experimental results and comparisons. Section "Conclusion" concludes and outlines future work.

## Preliminaries

### Multi-scroll chaotic map

Chaotic maps are frequently used in encryption since they can produce unpredictable pseudo random numbers. Consider a traditional single-scroll chaotic system (i.e., the Lorenz, Chen, or Chua system); these systems will have a single attractor whose chaotic behaviour will be limited to only that region in phase space. However, in contrast, multi-scroll chaotic maps can create many chaotic attractors; thus, the multi-scroll chaotic map can reach a more complex and random context. These characteristics make them ideal for secure encryption, image scrambling, and key generation^23^. In this article, a New 4D multi-scroll chaotic map is designed to enhance the security of image encryption. These multi-scroll maps are more random than single-scroll maps, characterised by higher entropy and complexity, Increased Key Sensitivity, and better uniform distribution. Consequently, it is difficult to predict the next state^24^.

Mathematical Representation of New Multi-scroll Chaotic Maps:

New Multi-scroll Chaotic Maps are created by modifying traditional chaotic systems^24^ using nonlinear functions (such as piecewise linear functions, step functions, or sine/cosine perturbations). Each term in these equations contributes to the chaotic behaviour and control parameter value for the New Multi-scroll chaotic map is a = 2.5; b = 8.5; c = 3; d = 0.0001; e = 1.75 is given in Eq. 1.

1 $$\begin{aligned} \dot{X} = & \;aY + bZ - W + f\left( X \right) \\ \dot{Y} = & \; - \tan \left( Y \right) + cZ + YZ \\ \dot{Z} = & \;ay + dZWX - ef\left( X \right) \\ \dot{W} = & \;bZ\tan \left( W \right) - eY \\ \end{aligned}$$

X, Y, Z, W—State variables that define the system dynamics.

a, b, c, d, e—Control parameters that determine the chaotic behaviour.

f(X)—Nonlinear function controlling the scroll formation.

The function f(X) controls the scroll formation, making it multi-scroll instead of single-scroll.

2 $$f\left( X \right) = X - \mathop \sum \limits_{n} sign \left( {X + n} \right) + 0.2 \sin \left( {2\pi X} \right)$$

Where:

- The sum of sign functions creates a piecewise discontinuous function, allowing the trajectory to switch between different scrolls.
- The sinusoidal term 0.2sin(2πX) introduces continuous oscillations to increase randomness.
- In Eq. 2 we use the fixed index set: n  $\epsilon$ {-12, -10, -8, -6, -4, -2, 0, 2, 4, 6, 8, 10, 12} (step size 2).
- Initial conditions are obtained using image adaptive key generation. (X_1_, Y_1_, Z_1_, W_1_)  $\epsilon$ [0,1). With (a, b, c, d, e) = (2.5, 8.5, 3, 0.0011, 1.75), dt = 0.005, and T= 256⨯256 steps (discarding the first 10% as transient), for the initial condition yielded the steady-state ranges: X $\epsilon$ [− 9.9762, 5.9224], Y $\epsilon$[− 0.5906, 0.5998], Z $\epsilon$ [− 0.4741, 0.4626], W $\epsilon$ [0.6110, 3.6080].

**Fig. 1:**
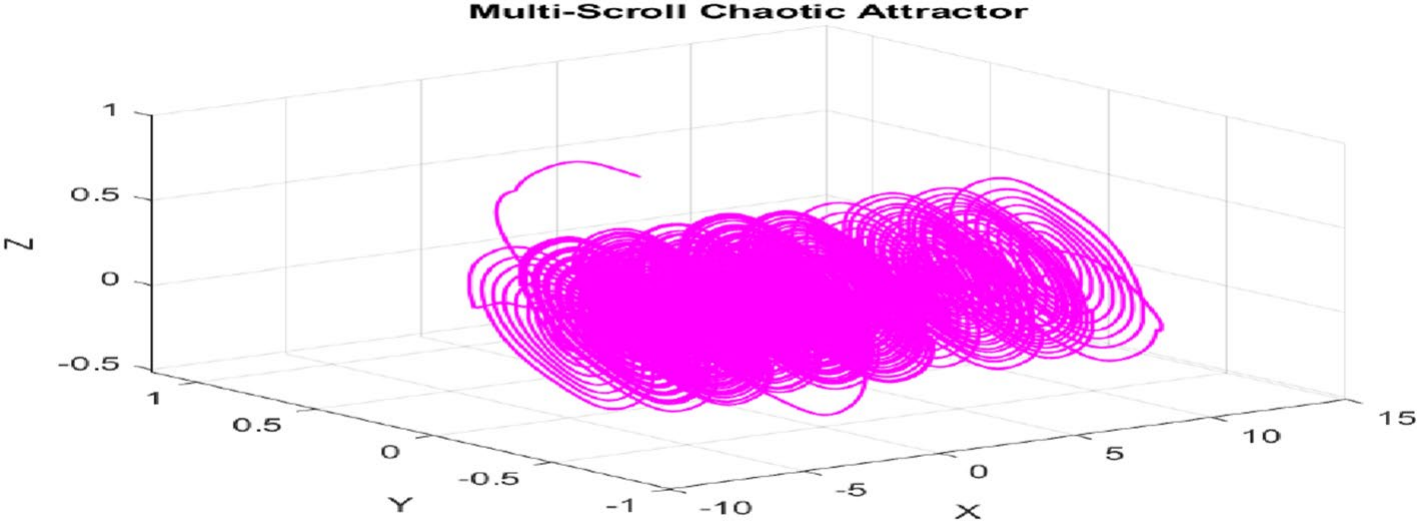
3D attractor diagram for new multi-scroll chaotic map.

**Fig. 2:**
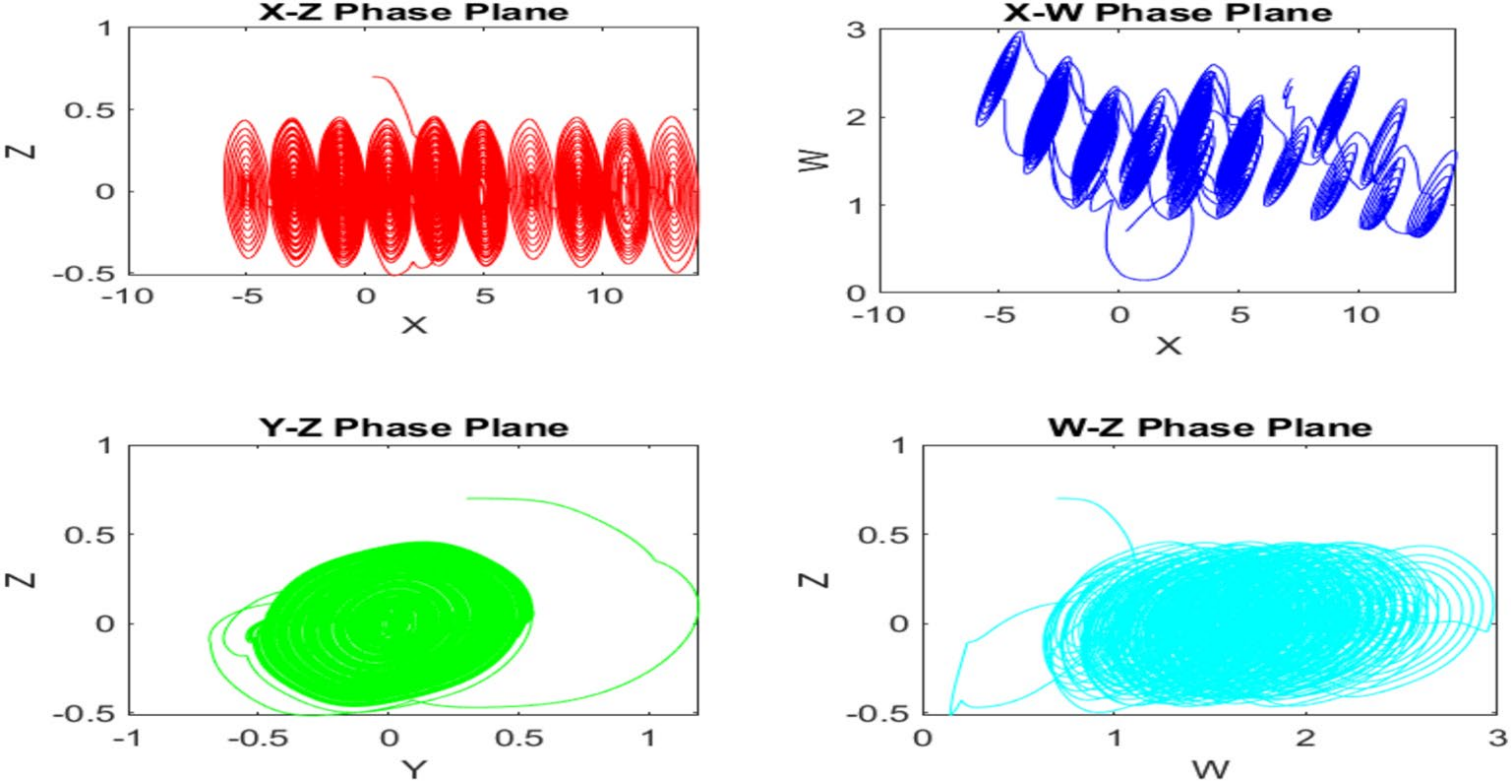
2D attractor diagram for new multi-scroll chaotic map.

**Fig. 3:**
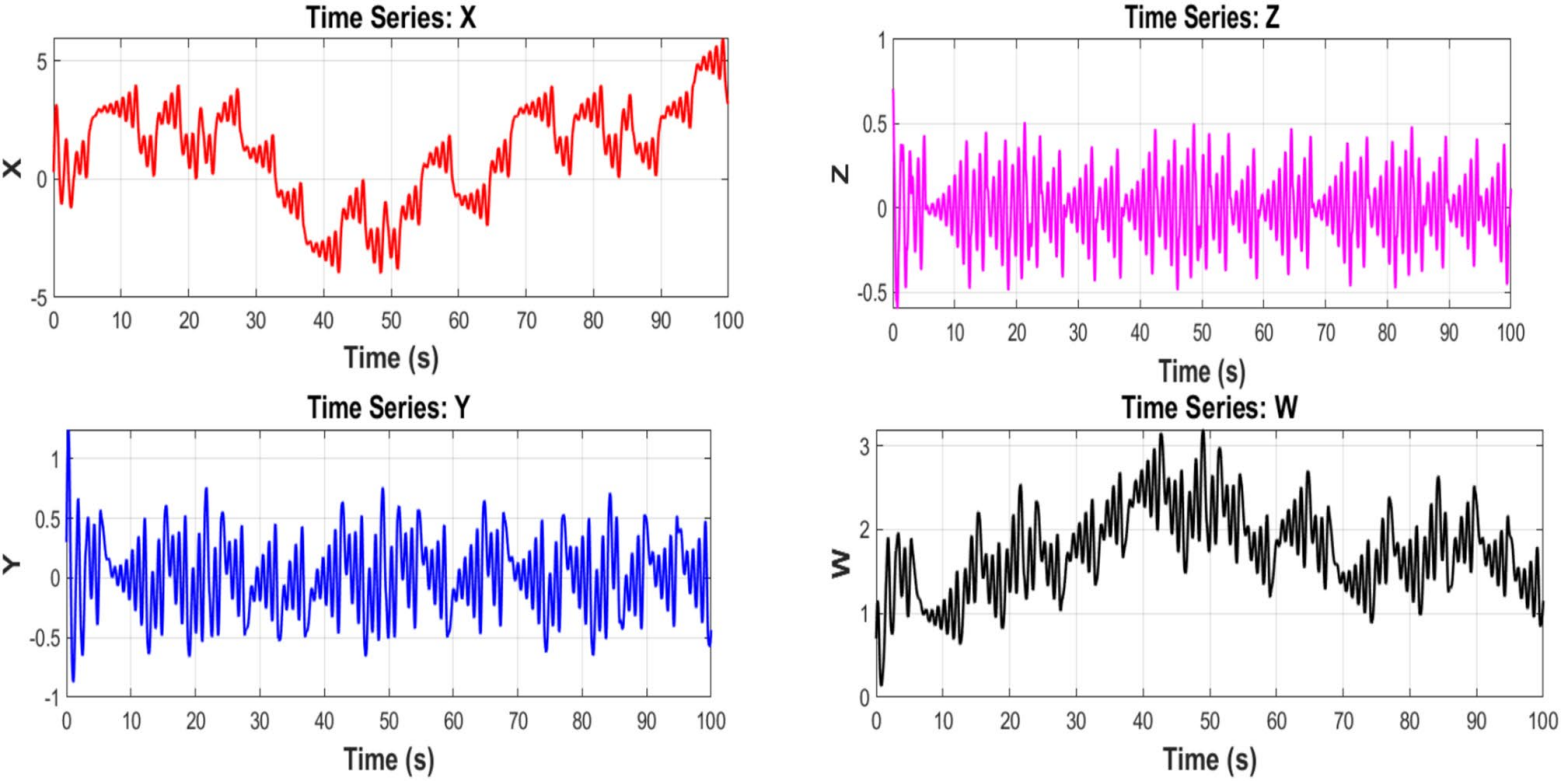
Time series diagram for new multi-scroll chaotic map.

The Figs. 1 and 2 displays the 3D and 2D Attractor diagram which shows rich dynamic characteristics of new 4D multi-scroll chaotic map. The time series plot of each chaotic sequence is demonstrated in the Fig. 3.

Multi-scroll attractor diagram -3D PLOT:

Multi-scroll attractor diagram -2D PLOT:

#### Lyapunov exponent

**Fig. 4:**
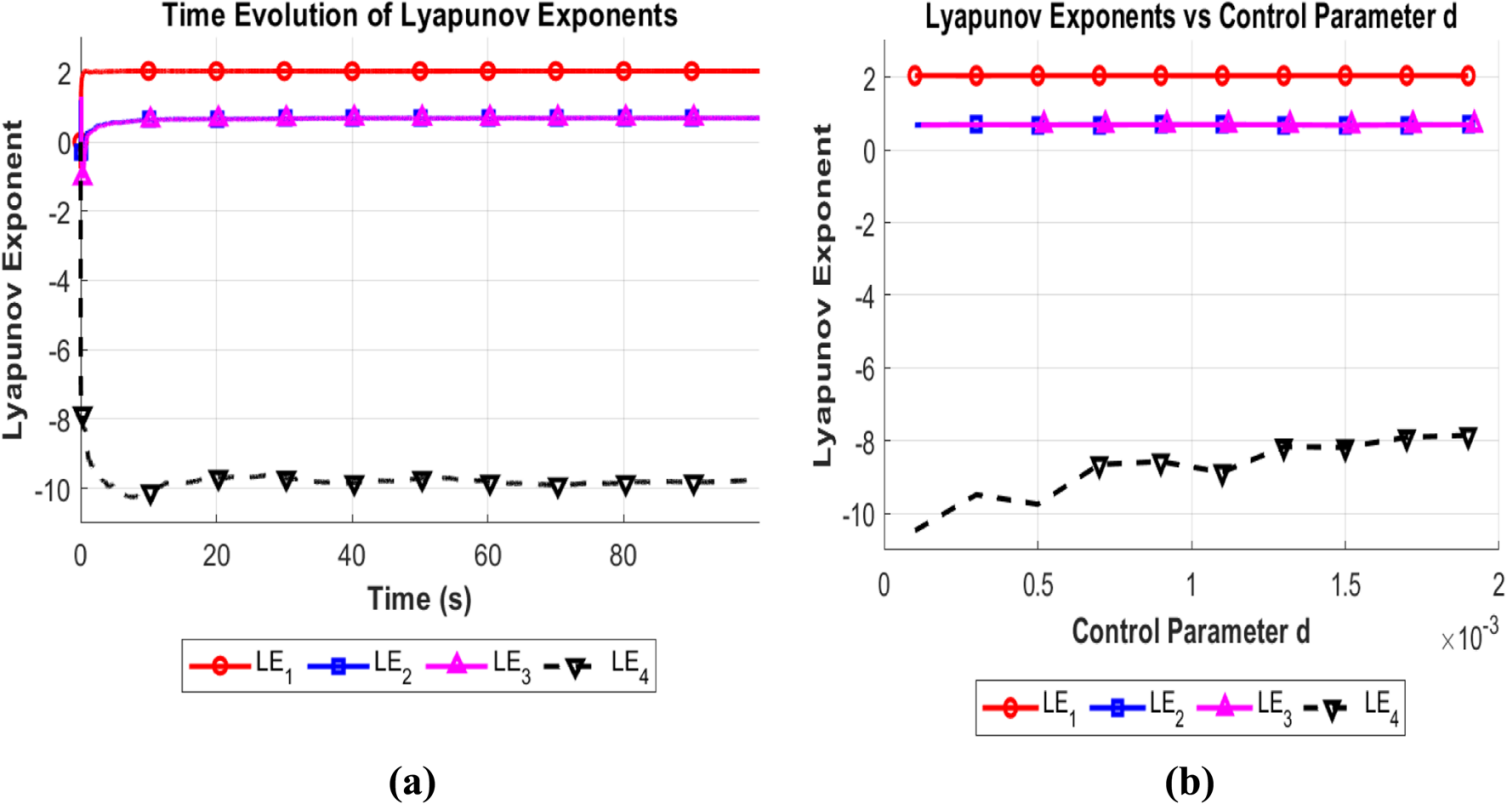
(**a**) Time evolution of Lyapunov exponents (**b**) Lyapunov exponent for varying ‘d’.

The Lyapunov exponent (LE) is one of the most crucial measures for assessing the sensitivity of a chaotic system to initial conditions. The LE defines the rate at which contiguous orbits diverge in a nonlinear dynamic map to evaluate statistical chaos. If the LE is positive, then the map will perform chaotically. It indicates that the map behaves in a hyperchaotic manner when there are two or more positive Lyapunov exponents^25^. For the control parameter, a = 2.5; b = 8.5; c = 3; d = 0.0019; e = 1.75, Fig. 4a and b shows the positive LE of the multi-scroll chaotic system. A 4D chaotic system enters a hyperchaotic state when it has two or more positive LE values. The Lyapunov exponent of the New multi-scroll chaotic map, for the initial conditions of X_1_ = 0.37569937711624, Y_1_ = 0.26398764600563, Z_1_ = 0.10739354407345, W_1_ = 0.44455963018465***.*** The LEs are computed using the variational equations derived from the Jacobian matrix of the system, given in Eq. 3

3 $$J= \left[\begin{array}{cccc}1 + 0.4\pi cos(2\pi X) & a& b& -1\\ 0& -se{c}^{2}\left(Y\right)+z & Y + c & 0\\ dZW - e(1 + 0.4\pi cos(2\pi X)) & a& dWX & dZX\\ 0& -e & b tan(W) & bZ / {cos^{2} } (W)\end{array}\right]$$

The Lyapunov exponents are calculated using the QR decomposition method over many iterations. The time evolution of LEs is obtained from Eq. 4

4 $$LE_{j} = { }\mathop {\lim }\limits_{{t{ } \to \infty }} \frac{{1{ }}}{t}{ }\mathop \sum \limits_{k}^{t} \log \left[ {\begin{array}{*{20}c} {R_{{j{ }j}} { }\left( K \right)} \\ \end{array} } \right]$$

Where:

- ${LE}_{j}$ is the j-th Lyapunov exponent
- ${R}_{j j} (K)$ is the j-th diagonal element of the R matrix obtained from QR decomposition of the product of Jacobian matrices over time
- t is the total number of steps (integration time)
- The Jacobian matrix is computed at each time step using the system’s equations

Where ${R}_{jj }\left(K\right)$ represents the diagonal elements of the R matrix obtained from QR decomposition at iteration K. The positive Lyapunov exponent represents the hyperchaotic nature of the system. The calculated Lyapunov exponents for the New Mult-Scroll chaotic system are

$${\text{LE}}1 = 2.0440{\text{, LE}}2 = 0.6940,{\text{ LE}}3 = 0.6933,{\text{ LE}}4 = - 7.8454$$

*Lyapunov-based chaotic analysis*

The dominating Lyapunov exponent of the proposed 4D multi-scroll system was compared with existing hyperchaotic systems to further verify its chaotic strength. The Lyapunov exponent, measures the sensitivity of a system with respect to its initial conditions. A positive dominating Lyapunov exponent, which conveys the intrinsic chaos and unpredictability. Also denotes a high divergence of close trajectories.

The prominent Lyapunov exponents of the suggested system and several benchmark chaotic systems from recent research^23,24,54–57^ are shown in Table 1. A dominant Lyapunov exponent of 2.0470 is attained by the suggested algorithm, which is noticeably higher than those in the comparison. It is validated that the proposed system is more suitable for safe image encryption tasks since it exhibits more substantial chaotic divergence. Key sensitivity, diffusion strength, and resistance to known-plaintext or differential attacks are all improved by a high Lyapunov exponent, which guarantees that even slight changes in the plaintext or key values will result in radically different ciphertexts. This comparison further supports the robustness and unpredictable nature of the suggested hyperchaotic structure.

**Table 1:** Comparative analysis of dominant Lyapunov indices.

Hyper chaotic system	REF ^23^	REF ^24^	REF ^54^	REF ^55^	REF ^56^	REF ^57^	Proposed algorithm
Dominant Lyapunov indices	0.136181	0.23635	0.07	1	0.0396	0.2	2.0470

#### Calculating the Kaplan-Yorke (Lyapunov Dimension (DKY))

The Kaplan-Yorke Dimension (DKY), also known as the Lyapunov Dimension^24^., estimates the fractal dimension of a chaotic system based on its Lyapunov exponents is given in Eq. 5. It helps us understand how complex the system’s attractor is.

5 $$D_{KY} = j + \frac{{\sum\nolimits_{i = 1}^{j} {LE_{i} } }}{{\left| {LE_{j + 1} } \right|}}$$

- j is the largest index where the sum of the first j Lyapunov exponents is still positive.
- LE_i_ are the Lyapunov exponents sorted in descending order.
- LE_j+1_ is the first negative exponent after summing the positive ones.

DKY Calculation for d = 0.0019

Find j (largest index where sum is positive)

- LE_1_ + LE_2_ + LE_3_ = 2.0440 + 0.6940 + 0.6933 = 3.4313
- Next exponent LE_4_ =  − 7.8454is negative. So, j = 3.

Compute DKY using the formula

$${D}_{KY}=3+\frac{\left(2.0440+0.6940+0.6933\right)}{\left|-7.8454\right|}$$

$${D}_{ky }=3+0.4374$$

$${D}_{KY}=3.4374$$

Interpretation of DKY (Kaplan-Yorke Dimension)

- D_KY_ = 3.4374 means that the chaotic attractor of multi-scroll system has a fractality between 3 and 4D.
- A higher D_KY_ indicates a higher degree of chaos and complexity, making the encryption more secure.
- Simple Lorenz system: D_KY_ ≈ 2.06 (less chaotic)
- Chen system: D_KY_ ≈ 2.4 (moderate chaos)
- Hyperchaotic systems: D_KY_ > 3 (stronger chaos)
- Hyperchaotic systems (DKY > 3) are harder to predict, making them ideal for cryptographic applications. Thus the Table 2 shows by varying the control parameter value ‘d’ the mutli scroll chaotic map results in the postive lyapunov exponent with all DKY value validates the system is hyperchaotic.

**Table 2:** Lyapunov exponent value and DKY dimension for varying control parameter “d”.

d	LE₁	LE₂	LE₃	LE₄	DKY dimension
0.0001	2.0457	0.6848	0.6839	− 10.4714	3.3261
0.0003	2.0440	0.6943	0.6936	− 9.4808	3.3620
0.0005	2.0463	0.6897	0.6889	− 9.7431	3.3515
0.0007	2.0470	0.6905	0.6899	− 8.6464	3.3964
0.0009	2.0447	0.6949	0.6941	− 8.5688	3.4007
0.0011	2.0439	0.6968	0.6962	− 8.8606	3.3879
0.0013	2.0442	0.6907	0.6899	− 8.1583	3.4198
0.0015	2.0465	0.6785	0.6778	− 8.1755	3.4162
0.0017	2.0470	0.6890	0.6883	− 7.8993	3.4335
0.0019	2.0440	0.6940	0.6933	− 7.8454	3.4374

#### Kolmogorov entropy analysis

**Fig. 5:**
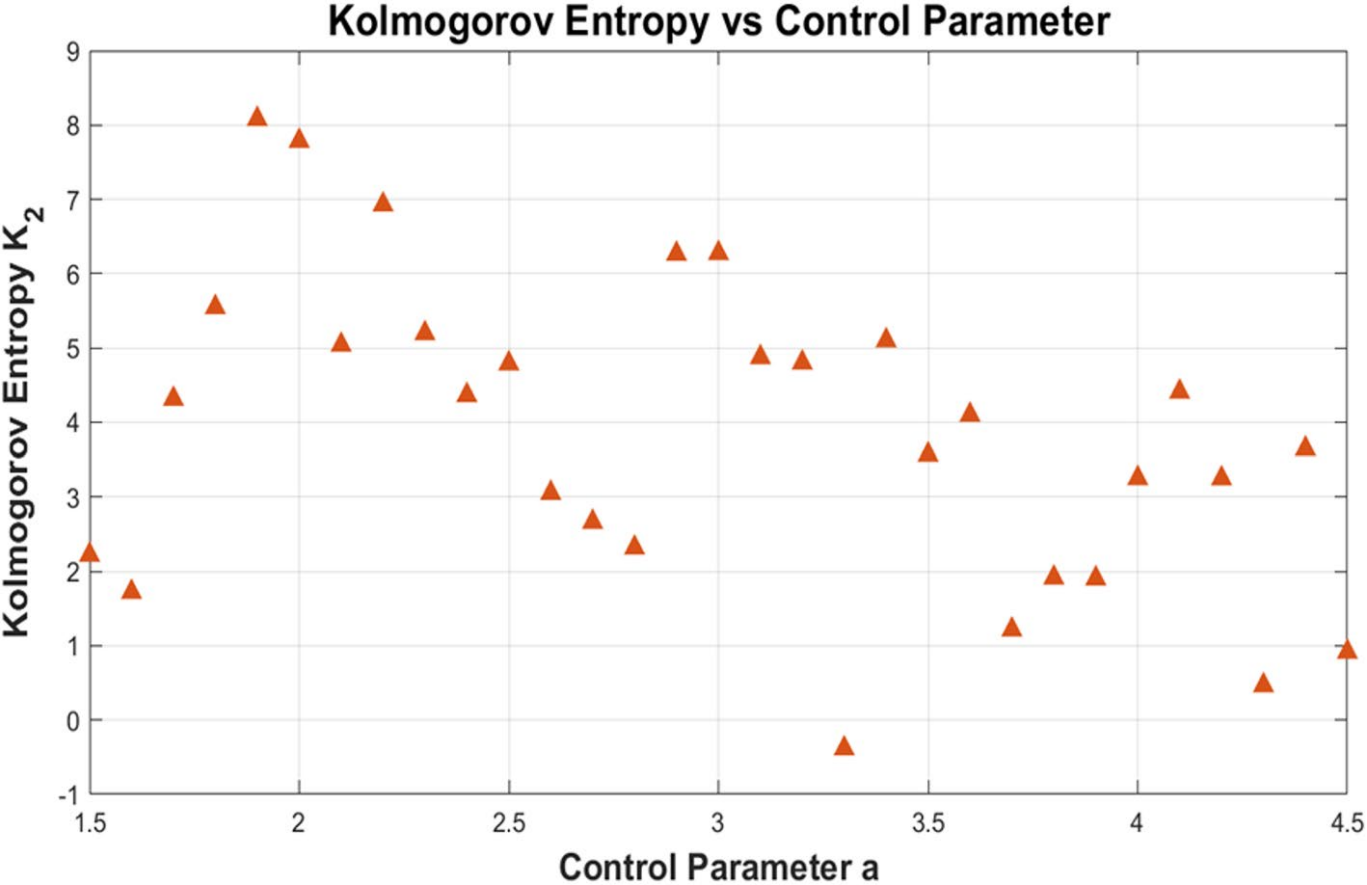
Kolmogorov entropy of multi scroll chaotic map.

Future trajectory of the non linear system can be estimated from its current state using the Kolmogorov Entropy (KE) indicator. A higher Kolmogorov Entropy in chaotic maps used for image encryption denotes a higher level of complexity and randomness, which is preferable for security purposes. If the KE is 0, then the nonlinear system is regular to the movement. A positive KE suggests that additional data is required for the nonlinear system to estimate the subsequent trajectory. A nonlinear system with a positive KE is thus considered unpredictable. A standard method for measuring KE is to find uncorrelated locations along a trajectory in phase space,and it makes easier to calculate KE value. The proposed 4D multi scroll chaotic map gives average Kolmogorov entropy of 3.5403 represents the enhanced entropy and nonlinearity. Figure 5 shows how Kolmogorov Entropy (K₂) changes with the control parameter a, highlighting sensitivity and complexity at different a value. Table 3 shows that the proposed encryption algorithm has the highest Kolmogorov Entropy (KE) value compared to other methods, indicating superior unpredictability, and enhanced randomness.

**Table 3:** Comparisons of Kolmogorov entropy.

Reference	Proposed system	Kolmogorov entropy
Proposed	New multi-scroll chaotic maps	3.5403
Ref ^51^	2D Salomon map	2.4624
Ref ^52^	2D Zettle map	2.5026
Ref ^40^	4D Non-degenerate chaotic system (4D-NDCS)	1.94
Ref ^53^	2D Price map	2.4530

#### Hyperparameter selection and tuning

To maximise the chaotic behaviour of the proposed 4D multi-scroll map, the initial conditions are adaptively extracted from EfficientNet-B3 features. At the same time, the key parameters are fixed at a = 2.5, b = 8.5, c = 3, d = 0.0001, and e = 1.75, based on a control parameter sweep over a ∈ [1.5,4.5]. Kolmogorov entropy and Lyapunov exponents were computed using a time series of 50,000 iterations (after discarding 10,000 transient steps), with numerical integration carried out using a time step of h = 0.001.A custom nonlinear function f(X), incorporating sign and sinusoidal terms, was designed to induce scroll-rich dynamics and boost system unpredictability—drawing conceptual parallels from the 2D-HELS hyperchaotic map^25^ and DNA-based spatiotemporal models. The chosen parameters yielded strong chaotic characteristics, as evidenced by positive Lyapunov Exponents and an average Kolmogorov Entropy of 3.5403. These methodical enhancements not only improve encryption robustness but also adhere to recognised principles of secure encryption design.

#### NIST test

The National Institute of Standards and Technology (NIST) SP 800-22, the most popular cryptography-focused randomness testing suite, consists of 16 tests that can identify numerous typical errors in non-random sequences. It provides p-values for every test to determine whether to reject the null hypothesis. These tests search for patterns to determine the likelihood that a particular sequence originated from a random source of patterns^26^. To test the sequence, we generate 5000 sequences with length of 2^20^. All of the NIST tests are passed if the p-value, which represents the likelihood of each test, is more significant than 0.01. The NIST test for the New 4D multi-scroll chaotic sequence is displayed in the Table 4 which shows that all the sequence p values are more significant than 0.01, concluding the randomness of the generated sequence.

**Table 4:** Results of the NIST SP 800-22 Test of new multi-scroll chaotic map.

S. NO	Statistical test	*P*-value				Status for randomness
		X	Y	Z	W	
1	Frequency test (Monobit)	0.3991001	0.3507686	0.4556475	0.0260142	Success
2	Block frequency test	0.5950266	0.7996856	0.9909122	0.9419595	Success
3	Run test	0.1958906	0.1569629	0.3068484	0.1613133	Success
4	Longest run test	0.1724787	0.1408724	0.1689116	0.4445189	Success
5	Binary matrix rank test	0.6676488	0.2105126	0.5339822	0.7046106	Success
6	FFT	0.2188213	0.5386642	0.4798148	0.1894331	Success
7	Non-overlapping template	0.9397471	0.1783955	0.9602249	0.4636701	Success
8	Overlapping template matching	0.1363661	0.4101915	0.8498319	0.2209427	Success
9	Universal	0.1575374	0.1427951	0.2362873	0.1456483	Success
10	Linear complexity test	0.5153266	0.5083476	0.4488440	0.7075788	Success
11	Serial test	0.7034016	0.1234249	0.9320031	0.1938909	Success
12	Approximate entropy test	0.8249837	0.9098061	0.1216421	0.2409390	Success
13	Cumulative sum test (Forward)	0.4209205	0.3322881	0.2105771	0.8080550	Success
14	Cumulative sum test (Backward)	0.5361926	0.5552235	0.5561310	0.5096615	Success
15	Random excursion	0.1533077	0.8041317	0.7605407	0.3368639	Success
16	Random excursion variant	0.8749470	0.9605150	0.1368606	0.5258622	Success

### EfficientNet-B3

**Fig. 6:**
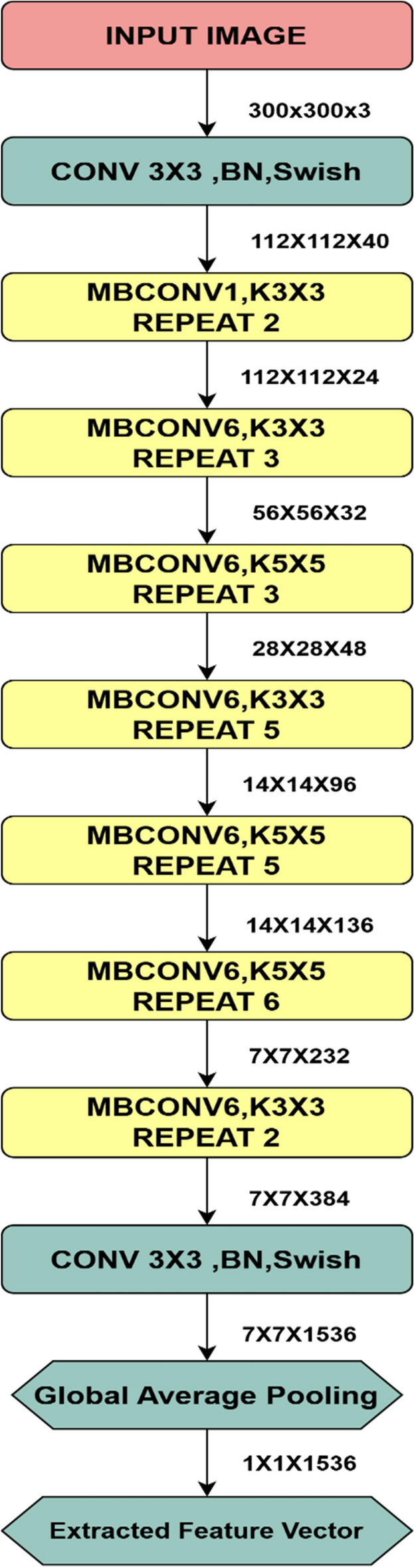
Architecture of EfficientNet-B3.

The proposed work uses a pre-trained EfficientNet-B3 Deep neural network to extract the features from each image, which is then transformed into unique initial conditions for the Proposed New multi-scroll chaotic map. The network is used only to extract the features from the convolutional layer, and the classification head of the network is removed. The EfficientNet-B3 is implemented in this algorithm because of its scaling capabilities in the depth, width and input resolution of the input data, and it has efficient compromise between the accuracy and computational resources utilized^27^. For the extraction of features, the network uses the Mobile inverted bottleneck convolution (MB Conv) block that uses inverted residual connections^28^. The final Global Average Pooling (GAP) layer reduces the feature vectors into flattened vectors that extract high-dimensional image semantics. The architecture of EfficientNet-B3 is shown in Fig. 6, and the featured vector output of the Global Average Pooling is used for further processing of initial conditions. The compound scaling method is the core innovation in the EfficientNet architecture using single scalar factor ϕ to uniformly scale all three dimensions such as depth, width and resolution given in the Equation is analysed in the paper^27^.

6 $${\text{Depth}}~\left( {\text{d}} \right) = \alpha ^{\phi } ;~{\text{Width}}~\left( {\text{w}} \right) = \beta ^{\phi } ;~{\text{Resolution}}~\left( {\text{r}} \right) = \Upsilon ^{\phi } {\text{ }}\alpha :\beta ^{2} :\Upsilon ^{2} \approx 2$$

Where:

$Upalpha$, $\upbeta$ and $\Upsilon$ are constants determined by small grid search.

**Fig. 7:**
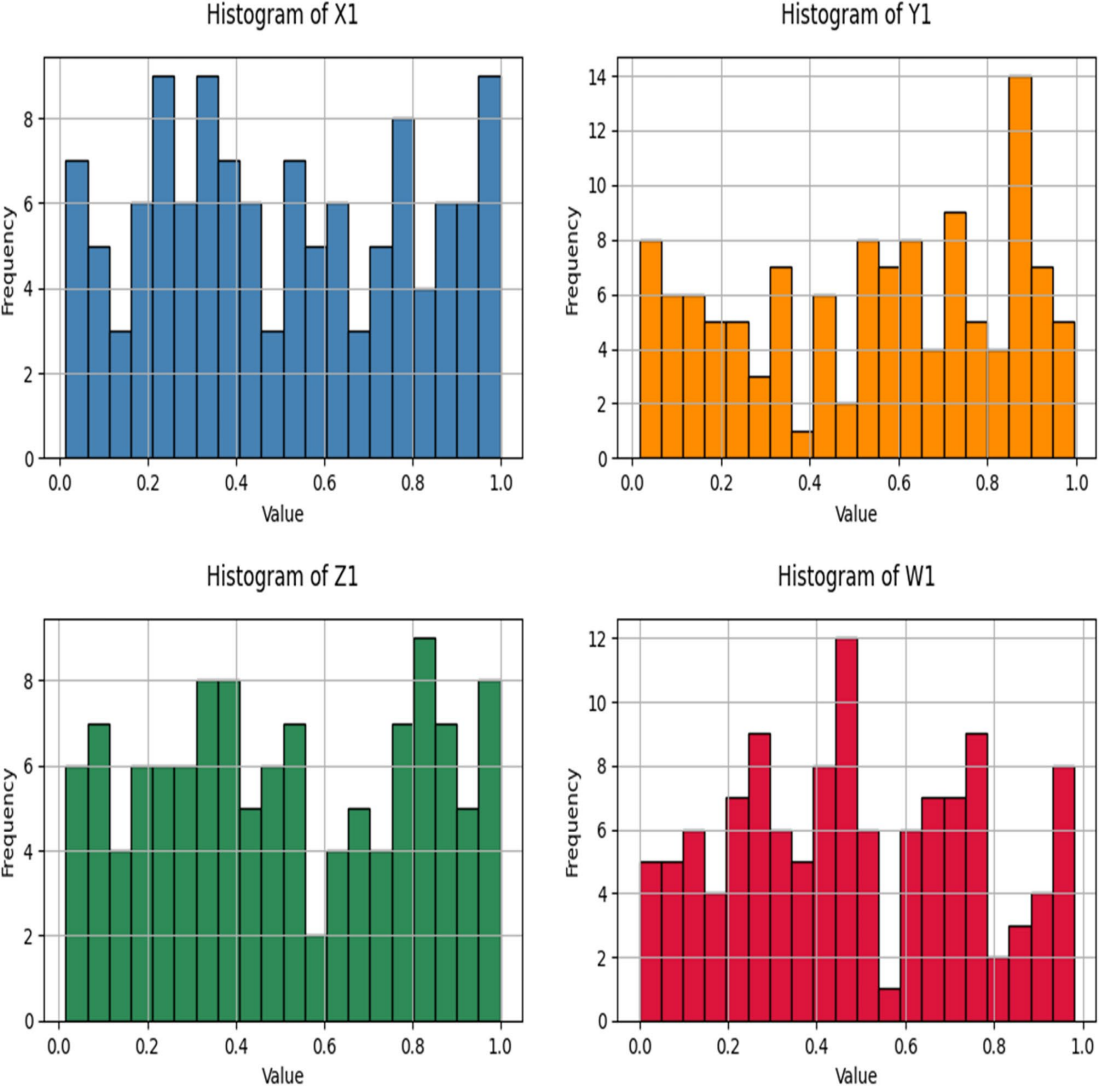
Histogram of initial conditions.

**Fig. 8:**
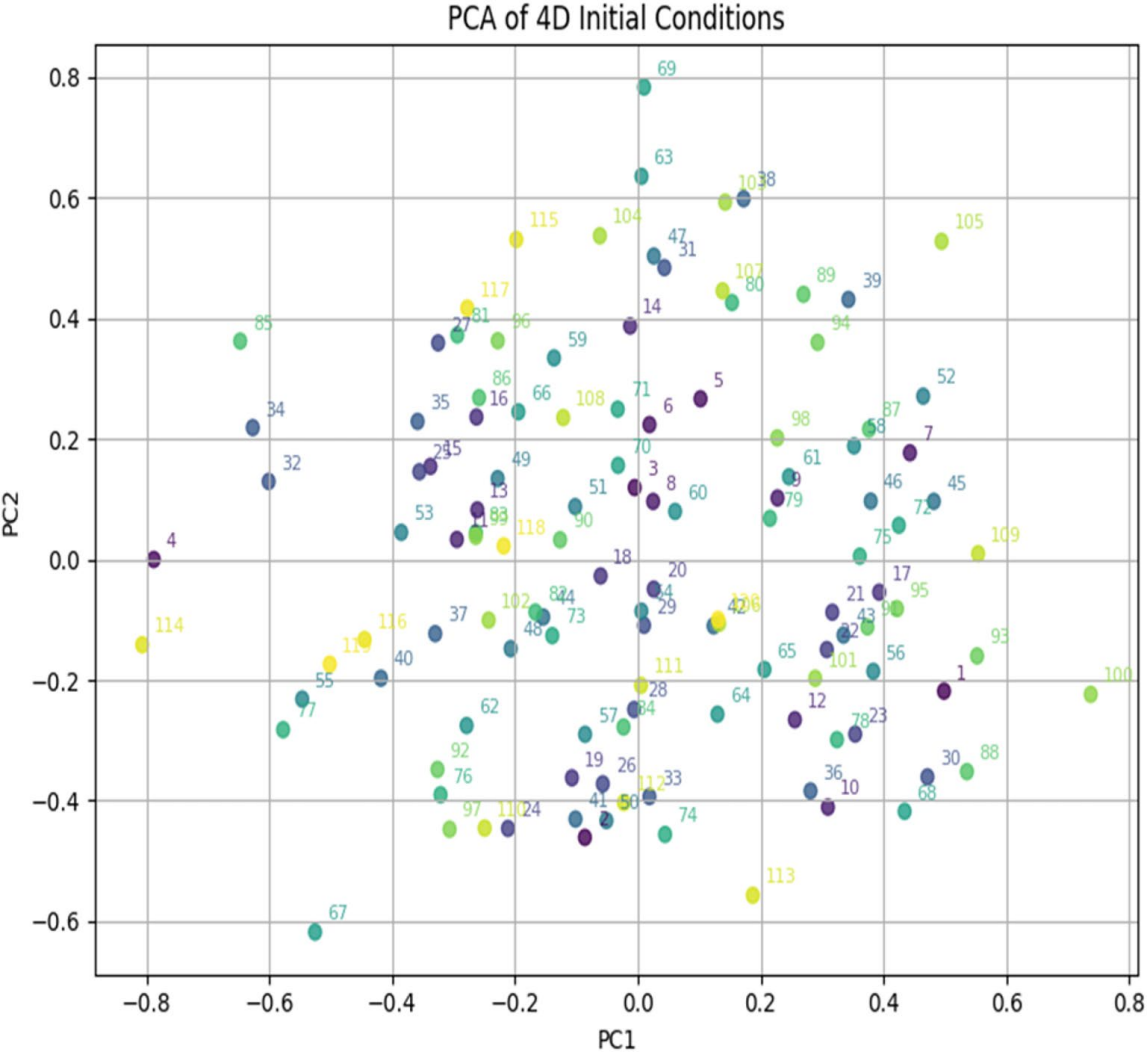
PCA analysis of initial conditions.

The high-dimension features extracted from the image are then given to the SHA256 hashing algorithm to get 64-bit integers, which are then normalized to the range of [0,1]. The four initial conditions are derived from the output hash function, which is given as a unique key value to the multi-scroll chaotic map. A sample of 120 images is taken from the class of natural data sets ^29^**,** and 15 images are taken in 8 classes to show the algorithm’s effectiveness. The histogram of initial conditions (X_1_, Y_1_, Z_1_, W_1_) for 120 sample images is given in Fig. 7. The distribution of the initial values for each image is unique, and it does not have a uniform distribution of the histogram plot. The histogram plot confirms that every image has a distinct initial condition, which is important in strengthening the security of the proposed method. The Principal Component Analysis is used to convert the 4-dimensional values into 2-dimensional values to analyze the scattering of the initial conditions. The PCA plot in Fig. 8 represents four initial conditions of a single image as a dot, and the distribution of initial values for different images indicates the uniqueness of the key value.

## Proposed algorithm

**Fig. 9:**
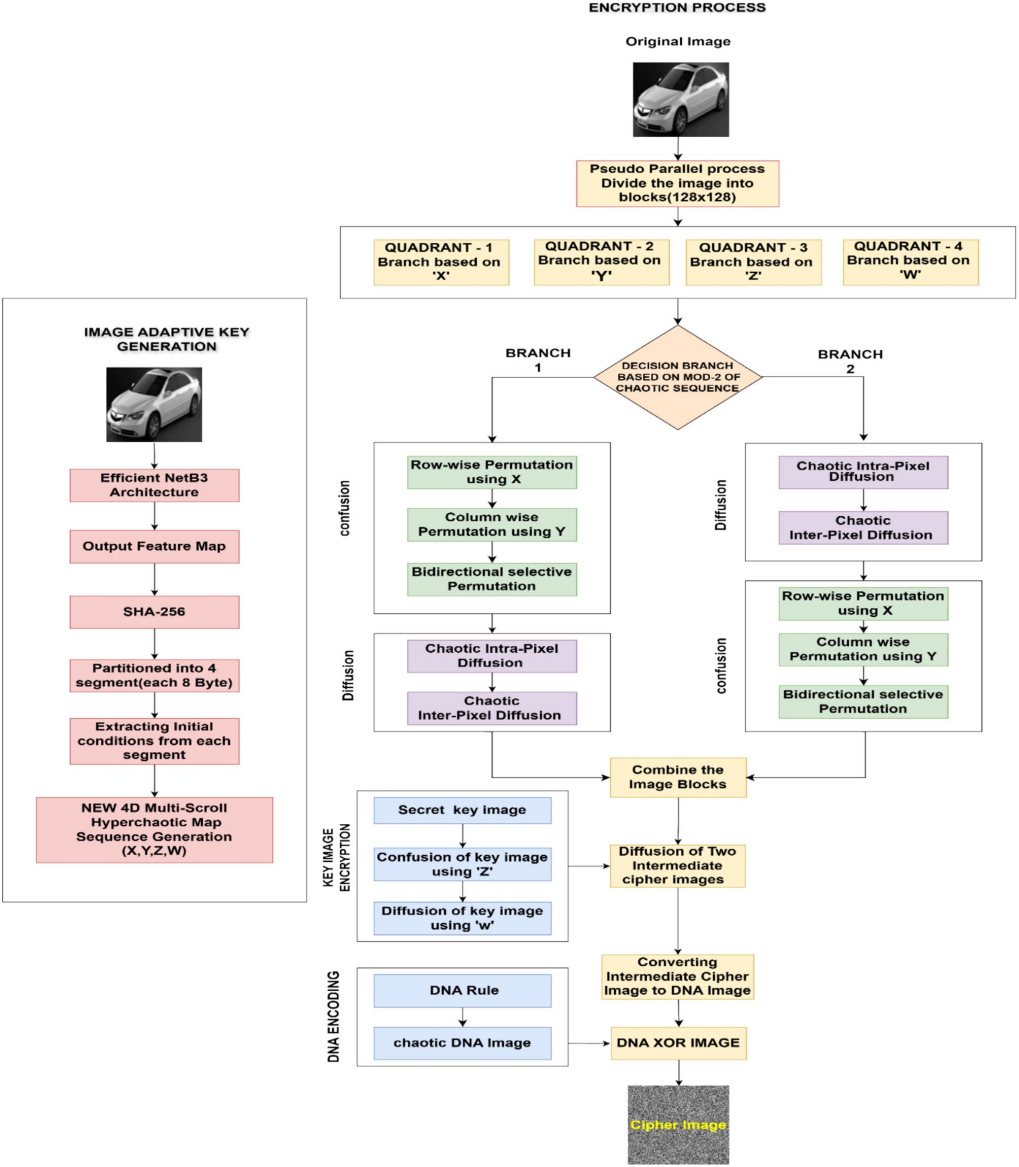
Block diagram for the proposed image adaptive encryption algorithm.

The proposed block diagram for image adaptive encryption using EfficientNet-B3 feature-guided multi-scroll chaotic map with modulo controlled pseudo-parallel branching is shown in the Fig. 9. The encryption algorithm effectively utilizes multiple stages of operation to implement an enhanced and robust solution for protecting different types of images. The algorithm contains five stages: image adaptive key generation, pseudo-parallel process selection, confusion-diffusion block, key image intermediate encryption, and finally, the dynamic DNA encoding stage. The steps to implement these five stages of the encryption algorithm are given below.

**Proposed Block Diagram**

### Encryption algorithm

**Step 1: Adaptive Key Generation**

EfficientNet-B3 is a well-balanced Convolutional Neural Network that uniformly scales depth, width, and resolution. It provides robust, discriminative features with moderate computational complexity and is pre-trained on large-scale RGB datasets. Channel replication forms a pseudo RGB image for grayscale images, allowing EfficientNet-B3 to extract meaningful features that can be normalized and scaled to serve as initial conditions in a chaotic encryption scheme. The EfficientNet-B3 gives feature vectors from each individual image.

- A grayscale image is represented as:
  $${\varvec{I}} \epsilon {\mathbb{R}}^{{\varvec{M}}{\varvec{X}}{\varvec{N}}}$$
- Channel Replication
  Pseudo RGB image is created by replicating the grayscale channel:
  $${{\varvec{I}}}_{{\varvec{R}}{\varvec{G}}{\varvec{B}}}\left({\varvec{i}},{\varvec{j}},:\right)=\left[{\varvec{I}}\left({\varvec{i}},{\varvec{j}}\right),{\varvec{I}}\left({\varvec{i}},{\varvec{j}}\right),{\varvec{I}}\left({\varvec{i}},{\varvec{j}}\right)\boldsymbol{ }\right];\boldsymbol{ }\boldsymbol{ }\boldsymbol{ }{{\varvec{I}}}_{{\varvec{R}}{\varvec{G}}{\varvec{B}}}\boldsymbol{ }\epsilon{\mathbb{R}}^{{\varvec{M}}{\varvec{X}}{\varvec{N}}{\varvec{X}}3}$$
- Feature Extraction Using EfficientNet-B3
  Feed ${I}_{RGB}$ into EfficientNet-B3 to obtain the feature vector:
  $$F = f(I_{{RGB}} )$$
- The feature vectors from EfficientNet-B3 are converted into bytes and then computed SHA-256 Hash to the raw bytes.
- The 32-byte hash is divided into 4 segments of 8 bytes (64 bits) and then each segment is normalized to the value of 0 to 1.
- Normalize the feature vector to [0,1]
  7 $${F}_{norm}= \frac{Integer value}{{2}^{64}- 1}$$

${F}_{norm}$ Obtained from each 4 segment is used as the initial condition values (X_1_, Y_1_, Z_1_, W_1_) for the multi-scroll chaotic map in the encryption process. Thus, we generate the image adaptive and unique initial conditions.

Confidentiality of the Encryption Key:

The encryption key is never sent in terms of raw form in the suggested technique. EfficientNet-B3 is used to extract deep picture features, which are transformed into a 256-bit value using the SHA-256 hash. The original properties or the key cannot be computationally extracted from the hash since SHA-256 is a one-way cryptographic method. Initial conditions for the 4D multi-scroll chaotic map are obtained by normalising the hash and splitting it into four 64-bit values. The key is kept secret throughout the encryption process, as all encryption processes are entirely powered by chaotic sequences generated from these initial conditions.

**Step 2: New Multi Sroll Chaotic Map**

- The initial conditions of the multi-scroll chaotic map are obtained from the feature map of the EfficientNet-B3. The image to be encrypted is given to EfficientNet to extract the output feature map, and the value is normalized to get the appropriate adaptive key value for each image. The multi-scroll chaotic map generates 4D complex chaotic sequence for the proposed algorithm.
- The generated chaotic sequence is used in pseudo-parallel process, confusion and diffusion blocks, key image diffusion and Dynamic DNA encoding to implement the high-performance encryption algorithm.

**Step 3: Pseudo-parallel Process**

- A unique pseudo-parallel processing approach is presented to improve the performance and preserve economical computational efficiency. This method performs well for simulating parallel execution for diffusion and confusion procedures.
- A 256 × 256 grayscale image is partitioned into four quadrants: Q1, Q2, Q3, and Q4, each with a resolution of 128 × 128 pixels.
- Based on the relevant multi-scroll chaotic value, each image quadrant dynamically chooses its operational branch. In particular, Branch 1 is executed if the modulo 2 of the chaotic value returns 0 (even); if not, Branch 2 is chosen for a modulo 2 results of 1 (odd).
  - Q1 selects its branch based on the chaotic variable X
  - Q2 selects its branch based on the chaotic variable Y
  - Q3 selects its branch based on the chaotic variable Z
  - Q4 selects its branch based on the chaotic variable W

- Branch 1 begins with the confusion phase followed by diffusion, whereas Branch 2 reverses this order, executing diffusion before confusion. This conditional branching introduces controlled randomness and directional variability, enhancing the overall security and unpredictability of the encryption scheme.
- The confusion module comprises three sequential permutation stages: Row-wise Permutation, Column-wise Permutation guided by Chaotic Sequences, and a Bidirectional Selective Permutation mechanism, each designed to disrupt pixel locality and introduce spatial complexity.
- The diffusion module consists of two hierarchical stages: Chaotic Intra-Pixel Diffusion (CIPD) and Chaotic Inter-Pixel Diffusion (CIPD), both aiming to propagate pixel intensity changes across local and global structures, thereby enhancing sensitivity to plaintext changes.
- A comprehensive explanation of the underlying mechanisms and operations within each stage of the confusion and diffusion modules is provided in **Step 4**.
- Different quadrants can select different branches in the same round since branch selection is done in each quadrant from its corresponding chaotic sequence. We consider this concept pseudo-parallel since quadrants address distinct data without cross-quadrant dependencies, thereby making them parallelizable on multi-core/GPU hardware or able to be processed sequentially with similar results.
- The decision made by the MOD-2 branch is deterministic. With fixed scaling *M* and fixed indexing, the branch bit *b* = ⌊∣(*k*)∣⋅*M*⌋ mod 2 is calculated from the key-driven chaotic sample for each quadrant. The same chaotic sequence is recreated during decryption with the identical initial conditions, parameters, time step, and indexing. The branch map exactly replicates similar to the original map. Next, we use the inverted sequence of operations: decryption does inverse diffusion first, then inverse confusion, and encryption does confusion first, then diffusion, and vice versa. There is no need to send any more metadata, like a branch map or permutation indices.

**Step 4: Confusion Block**

**Row-Wise Permutation**

In row-wise permutation, each row of the image is scrambled independently using a chaotic sequence. Since each row contains N pixel values (where N is the number of columns), we generate a chaotic sequence of length N for each row. This sequence acts as a guide for shuffling pixel positions. This reordering changes the position of pixels while preserving their values, adding confusion in the horizontal (row) direction.

Permutation Procedure:

Let the grayscale image be represented as a 2D matrix:

$$I\epsilon{\mathbb{R}}^{MXN}$$

where M is the number of rows and N is the number of columns (pixels per row).

For each row i ∈ {1, 2,.., M}, the permutation process is defined as follows:

1. Generate a chaotic sequence

8 $${X}^{(i)}={[x}_{1}^{(i)},{x}_{2}^{(i)},\dots ,{x}_{N}^{\left(i\right)}]$$

2. Sort the chaotic values in ascending order

9 $${p}^{(i)}= argsort ({x}^{\left(i\right)})$$

where ${P}^{(i)}=$ [${p}_{1}^{(i)},{p}_{2}^{(i)},\dots .,{p}_{N}^{\left(i\right)}]$ contains the indices that would sort ${x}^{\left(i\right)}$ in ascending order. x(i) denotes the chaotic sequence generated for the iii-th row of the image, and ${P}^{(i)}$ is the corresponding permutation index vector obtained by sorting ${{\varvec{x}}}^{\left({\varvec{i}}\right)})$ in ascending order. The superscript (i) refers to the row index in the image matrix.

3. Permute the pixel positions in row i

Let ${I}^{i}=\left[I\left(i,1\right),I(I,2,\dots ,I(i,N)\right]$ be the original pixel values in row i. The permuted row ${\widehat{I}}_{i}$ is computed as

10 $${\widehat{I}}_{i} \left(j\right)= {I}_{i}({p}_{j}^{\left(i\right)})$$

4. Construct the permuted image row-wise

11 $${\widehat{I} (\text{i},:)=\widehat{I}}_{i}$$

Repeat the above steps for all I ∈ {1, 2…, M} to construct the row-wise permuted image $\widehat{I}$.

Example:

The row of pixel values is:
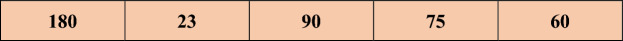


Generated chaotic values (same length as row):
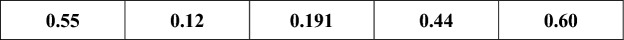


Sorted chaotic values:
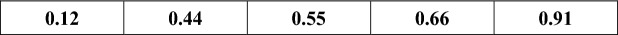


Sorted indices:
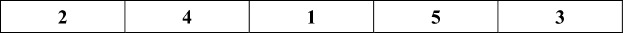


Permuted row:
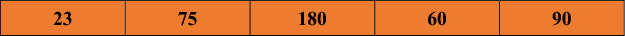


**Column-Wise Permutation:**

In column-wise permutation, we treat each column of the image separately and rearrange its pixel values vertically. Since each column has M pixel values (where M is the number of rows), we generate a chaotic sequence of length M per column. This process introduces vertical confusion in the image.

Permutation Procedure:

Let the grayscale image be represented as a 2D matrix:

$${\varvec{I}}\epsilon{\mathbb{R}}^{{\varvec{M}}{\varvec{X}}{\varvec{N}}}$$

where M is the number of rows and N is the number of columns (pixels per row).

For each row j ∈ {1, 2,.., N}, the permutation process is defined as follows:

1. Generate a chaotic sequence

12 $${Y}^{(j)}=[{y}_{1}^{\left(j\right)},{y}_{2}^{\left(j\right)},\dots .,{y}_{M}^{\left(j\right)}]$$

2. Sort the chaotic values in ascending order

13 $${Q}^{(j)}= argsort ({y}^{\left(j\right)})$$

Where ${Q}^{(j)}=$ [${q}_{1}^{(j)},{q}_{2}^{(j)},\dots .,{q}_{M}^{\left(j\right)}]$ contains the indices that would sort ${x}^{\left(i\right)}$ in ascending order. ${{\varvec{y}}}^{\left({\varvec{j}}\right)}$ is the chaotic sequence generated for the j-th column, and ${{\varvec{Q}}}^{({\varvec{j}})}$ is the corresponding permutation index vector obtained by sorting ${{\varvec{y}}}^{\left({\varvec{j}}\right)}$ in ascending order. The superscript (j) denotes the column index in the image matrix.

3. Permute the column pixels

Let ${I}^{j}={[I(1,j),I(2,j),\dots ,I(M,J)]}^{T}$ be the pixel values of column j. The permuted column ${I}^{j}$ is computed as:

14 $${I}^{j} \left(i\right)= {I}_{i}({Q}_{i}^{\left(j\right)},j)$$

4. Construct the permuted image

15 $$\widehat{I} \left(:,\text{j}\right)={\widehat{I}}^{j}$$

Repeat the above steps for all columns j 1 to N to obtain the fully column-permuted image $\widehat{I}$.

Example:
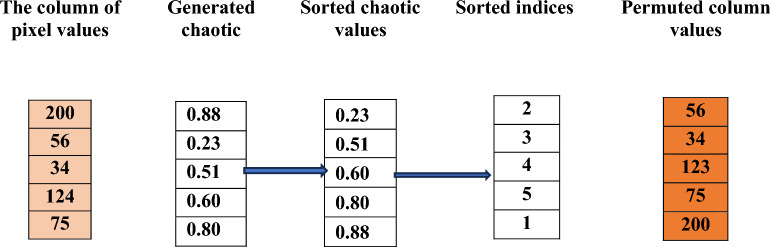


**Bidirectional Selective Permutation**

1. Generate chaotic values for each row (X_i)_

2. Quantize each chaotic value:

16 $$Q\left( i \right) = int(x\left( i \right) \times 255$$

3. Compute the Chaotic Position Index (CPI): sorted indices of Q

4. For each pair (${\beta }_{i}$ and ${\beta }_{i+1}$) Using CPI, calculate the Content Driven Index point (CDI)
  Compute the Mean and Entropy of the pixel pair.
  Calculate:
    17 $${CDI}_{1 }=max(1, \left|{\beta }_{i}\text{ X }{\beta }_{i+1}+\text{H X }100\right|mod 255)$$
    18 $${CDI}_{2 }=\text{max}(1, \left|{\beta }_{i}- {\beta }_{i+1}+\text{mean}\right|mod 255)$$
  If CDI₁ > CDI₂, swap pixel values at ${\beta }_{i}$ and ${\beta }_{i+1}$

5. Continue this process across all rows. Take the next adjacent pair from the sorted quantized chaotic values as ${\beta }_{i}$ and ${\beta }_{i+1}$

Example:

Chaotic Row (float values):
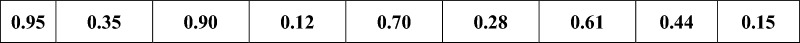


Quantized Chaotic Values (each = int (value × 255)):
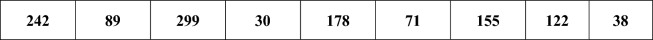


Original Pixel Row:
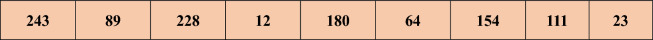


Generate CPI (Chaotic Position Index):

Sort quantized chaotic values and their original indices:
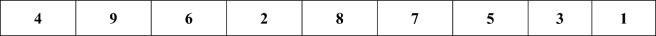


Select ${\beta }_{i}$ and ${\beta }_{i+1}$ using first two positions in CPI

${\beta }_{i}$= pixel at position 4 → 12

${\beta }_{i+1}$= pixel at position 9 → 23

- Calculate Mean of ${\beta }_{i}$ and ${\beta }_{i+1}$

$$Mean= \frac{12+23 }{2}=17.5$$

- Calculate Entropy H (Shannon Entropy)

Two unique values (12 and 23), each with probability = 0.5

$$H=[0.5 {log}_{2}\left(0.5\right)+ 0.5 {log}_{2}\left(0.5\right)] =1.0$$

- Compute Content-driven Index CDI_1_ and CDI_2_

$$\begin{aligned} CDI_{1 } = & \;max\left( {1, \left| {\beta_{i} {\text{ X }}\beta_{i + 1} + {\text{H X }}100} \right|mod 255} \right) \\ = & \;{\text{max}}\left( {1,\left| {12 X 23 + 1X 100} \right|mod 255} \right) \\ = & \;\max (1,\left| {376} \right|mod 255)) = 121 \\ \end{aligned}$$

$$\begin{aligned} CDI_{2} = & \;max\left( {1,\left| {\beta _{i} - \beta _{{i + 1}} + {\text{mean}}} \right|mod255} \right. \\ & \;\left. { = max\left( {1,\left| { - 11 + 17.5} \right|mod255} \right)} \right) \\ & \; = max\left( {1,6.5} \right) = 6 \\ \end{aligned}$$

- Decide to Swap

Since ${CDI}_{1}$ > ${CDI}_{2}$ (121 > 6), The swap is performed.

Swap values at positions 4 and 9:

Row Before:
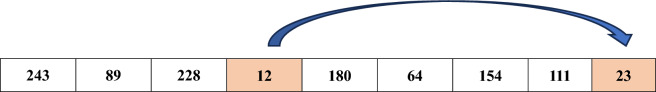


Row After selective permutation:
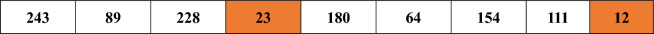


Summary

**Table:** 

Metric	Value
${\beta }_{i}$	12 (from position 4)
${\beta }_{i+1}$	23 (from position 9)
Mean	17.5
Entropy	1.0
${CDI}_{1}$	121
${CDI}_{2}$	6
Swap	YES ${CDI}_{1 }>{CDI}_{2}$

- Take the next adjacent pair from the sorted quantized chaotic values and fix the corresponding pixel values as ${\beta }_{i}$ and βᵢ₊₁ for the next iteration and continue this process across all rows.

Example next Iteration continues as:

${\beta }_{i}$ = pixel at position 6 → 64

$\beta _{{i + 1}}$ = pixel at position 2 → 89

**Step 5: Diffusion Block**

**Chaotic Intra-Pixel Diffusion (CIPD):**

Algorithm Steps

1. Generate Chaotic Value for Pixel

Obtain a chaotic float W​(i) ∈ (0,1) from your chaotic system.

2. Extract 4-bit Key:

Scale and reduce the chaotic value to obtain a 4-bit key

19 $$Q\left(i\right)= \left|W\left(i\right) X {10}^{15}\right| mod 16$$

This yields an integer in the range 0–15 (i.e. 4 bits).

3. Determine Transformation Type:

Further reduce $Q\left(i\right)=$modulo 4 to choose one of four operations for the Least Significant Half Segment:

20 $$\begin{gathered} T\left( i \right) = Q\left( i \right)mod4 \hfill \\ T\left( i \right)\left( {0,1,2,3} \right) \hfill \\ \end{gathered}$$

- $W\left( i \right)$ is the chaotic float value in the range (0,1) generated for the i^th^ pixel using your chaotic system.
- $Q\left( i \right)$ is 4-bit key (used for diffusion)
- $T\left( i \right)$ is the transformation type for Least Significant Half Segment operations

4. Convert the Pixel to Binary and Split into upper segment Most Significant Half Segment (MSHS) and a lower segment Least Significant Half Segment (LSHS)
  For an 8-bit pixel P(i) (range 0–255):
  Convert P(i) to its 8-bit binary form.
  Split into two 4-bit segments:
    Most Significant Half Segment (MSHS): Bits 7–4
    Least Significant Half Segment (LSHS): Bits 3–0

5. Diffuse the Most Significant Half Segment:

Use the entire 4-bit key $Q\left(i\right)$ to diffuse MSHS using XOR

21 $$Ne{w}_{\text{MSHS}}=\text{MSHS}\oplus Q\left(i\right)$$

**Diffuse the Least Significant Half Segment (LSHS)** on** T(i):**

Apply a transformation to LSHS based on T(i):

- **If T(i) = 0:** Set mask = Q(i) and compute:

22 $$Ne{w}_{\text{LSHS} }=\text{LSHS} \oplus Q(i)$$

- **If T(i) = 1:** Set mask = LROT(Q​(i), 1) (left rotate by 1 bit) and compute:

23 $$Ne{w}_{\text{LSHS }}=\text{LSHS }\oplus LROT(Q\left(i\right),1)$$

- **If T(i) = 2:** Set mask = RROT(Q5(i), 1) (right rotate by 1 bit) and compute:

24 $$Ne{w}_{\text{LSHS }}=\text{LSHS }\oplus RROT(Q\left(i\right),1)$$

- **If T(i) = 3:** Set mask = Complement(Q(i) (i.e. 15 − Q(i)) and compute:

25 $$Ne{w}_{\text{LSHS }}=\text{LSHS}\oplus Complement (Q\left(i\right))$$

Then perform:

26 $$Ne{w}_{\text{LSHS} }=\text{LSHS} \oplus mask$$

6. Recombine the MSHS and LSHS:

Form the new 8-bit pixel:

27 $$New Pixel=Ne{w}_{\text{MSHS }}| Ne{w}_{\text{LSHS}}$$

**Processing Each Pixel**

The algorithm iterates through each pixel in the image. For each pixel:

- Its chaotic key is computed.
- The 8-bit pixel is converted to binary and split into two 4-bit segments
- The **Most Significant Half Segment (MSHS)** is diffused using Q​(i) (via XOR).
- The **Least Significant Half Segment (LSHS)** diffused based on T(i) (using one of the four operations).

Example:

The original pixel value **201** is changed using Chaotic Inter-Pixel Diffusion (CIPD)

Original Pixel
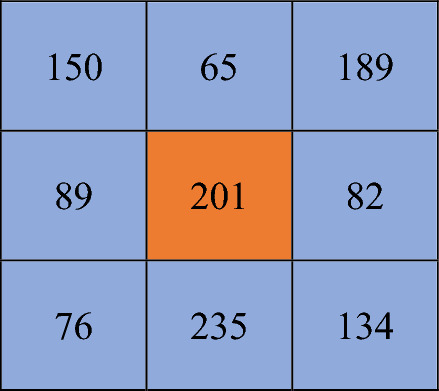


8-BIT Binary Value of Original Pixel Value:
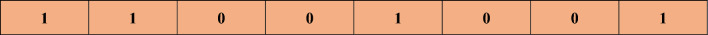

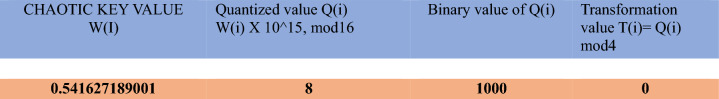


New Most Significant Half Segment (MSHS):
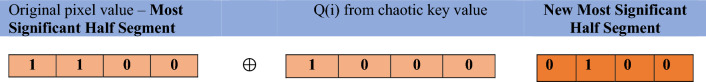


New Least Significant Half Segment calculated based on T(i) = 0:
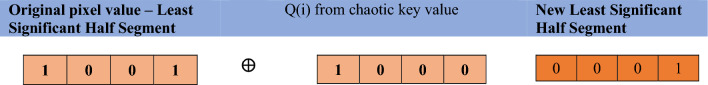


New 8-bit pixel value:
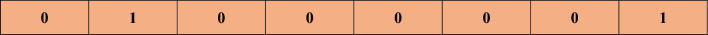



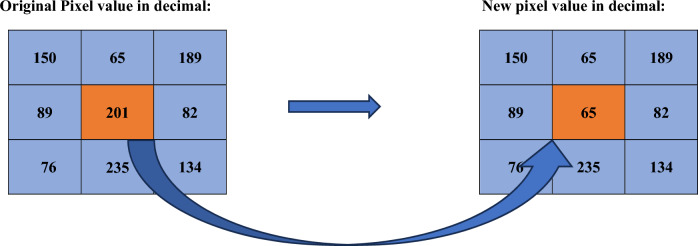


Chaotic Inter-Pixel Diffusion (CIPD):

- Input the Secret Key (e.g., “mySecret123”).
- Generate SHA-256 Hash of the key.
- Convert Hash to Decimal and then Modulo 255 to obtain the initial diffusion value D_0​_
- Flatten the image into a 1D pixel array $P=[{p}_{1},{p}_{2},\dots , {p}_{N}$]
- Generate a chaotic sequence $C=[{c}_{1},{c}_{2},\dots , {c}_{N}$]of the same length as the number of pixels (modulo 255).
- Perform pixel-wise diffusion using
  28 $${D}_{i=}({p}_{i}\oplus {D}_{i-1})\oplus {c}_{i}$$
- Reshape the 1D output back into a 2D image (or quaternion/RGB if required).

Example:
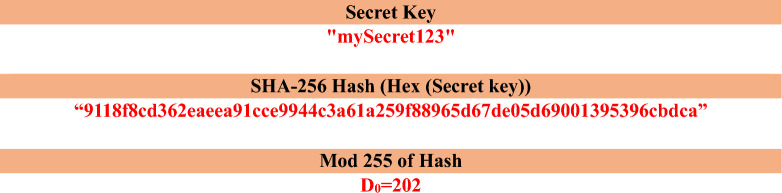


**New Diffused value using secret hash with previous diffused value:**

Step-by-Step Calculation:

$${D}_{i=}({p}_{i}\oplus {D}_{i-1})\oplus {c}_{i}$$

1. D_1_​ = (120 ⊕ 202) ⊕ 55 = 178 ⊕ 55 = 141​
2. D_2_​ = (45 ⊕ 141) ⊕ 190 = 164 ⊕ 190 = 26​
3. D_3_​ = (230 ⊕ 26) ⊕ 76 = 252 ⊕ 76 = 176​
4. D_4_​ = (12 ⊕ 176) ⊕ 30 = 188 ⊕ 30 = 158​

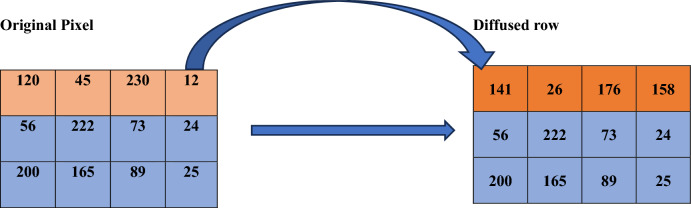


**Step 5: Key Image Diffusion**

- The quadrant blocks are processed through Branch 1 and Branch 2, completing the first level of the encryption stage.
- All the quadrant blocks of size 128 × 128 are merged to reconstruct a 256 × 256 image.
- This first-stage encrypted image of size 256 × 256 is further diffused using a key image to enhance security.
- A second-level diffusion acts as a global transformation, spreading the impact of any small change in the original image or key across the entire image.
- Provides avalanche effect, which is crucial for high security, and protects against known-plaintext and chosen-plaintext attacks

**Step 6: DNA Chaotic Image Diffusion**

- The final encrypted image is obtained by diffusing the output from Step 6 using a DNA-encoded chaotic sequence image of size 256 × 256. The output of the previous step is first converted into a DNA-encoded image using the Table 5, which is then XORed with the DNA chaotic sequence image using Table 6.
- Applying diffusion using a DNA chaotic sequence image at the final stage ensures that even a slight change in the original image or key leads to a completely different encrypted output. This enhances the avalanche effect, which is critical for strong encryption.
- The chaotic sequence image acts as a key-dependent diffusion layer, introducing high non-linearity and sensitivity to initial conditions, making brute-force and statistical attacks more difficult.
- Final diffusion increases the uniformity of the histogram, reducing clues about pixel intensity distribution and making ciphertext analysis ineffective.

**Table 5:** DNA rule.

DNA rule	00	11	10	01
1	A	T	G	C
2	A	T	C	G
3	T	A	G	C
4	T	A	C	G
5	G	T	A	C
6	G	T	C	A
7	C	T	A	G
8	C	T	G	A

**Table 6:** DNA XOR rule.

DNA base1	DNA base 2	DNA XOR
A	A	A
A	T	T
A	G	G
A	C	C
T	A	T
T	T	A
T	G	C
T	C	G
G	A	G
G	T	C
G	G	A
G	C	T
C	A	C
C	T	G
C	G	T
C	C	A

### Decryption algorithm

The chaotic sequences for *T* = 256 × 256 steps are regenerated by the decrypted using the same parameters, step size, iteration count, and indexing, as well as the same initial values (X_1_, Y_1_, Z_1_, W_1_). It replicates the same branch selection as in encryption by calculating the MOD-2 branch bit for each quadrant (Q1–Q4) from the associated chaotic sample (X, Y, Z, or W).

Within each quadrant, every operation is bijective and applied in reverse order.

- *DNA step* use the same rule to convert the DNA image back to bytes and use the same lookup table to reverse DNA-XOR.
- *Final XOR* Use the chaotic values obtained from the Z sequence to reverse the previous XOR.
- *Key-image mixing* use the Z and W sequences to regenerate the second-path term from the constant image, then cancel it using XOR to isolate the main path.
- *Diffusion* XOR the same chaotic values (from the Y or W sequence, depending on the branch) once to reverse pixel diffusion.
- *Confusion* use the inverse permutation (row-wise, column-wise, and bidirectional stages inverted) that was derived from the sort order that was formed from the X or Z sequence.
- *Reassembling* combine the four 128 × 128 blocks to create a 256 × 256 picture.

*Reversibility* There is a clearly defined inverse for every operation. When using the same mask, XOR is self-inverse. By employing its inverse index map, every permutation can be reversed. The original value is returned when a complement is applied twice. When the same rule and table are used, DNA encoding and DNA-XOR can be reversed. There is no need to send extra side information like branch maps or permutation indices because the branch decision, masks, and indices are created from the key-derived chaotic sequences. The original image can be retrieved precisely by decryption with the identical numerical settings.

## Results and performance analysis

The proposed multistage encryption algorithm integrates EfficientNet-B3 with a New 4D multi-scroll chaotic map and the algorithm is implemented using natural dataset images. The image adaptive key generation is executed using the python platform and the simulation analysis is carried out in MATLAB-R2024 on a PC Intel Core I5 with 16 GB RAM.

**Fig. 10:**
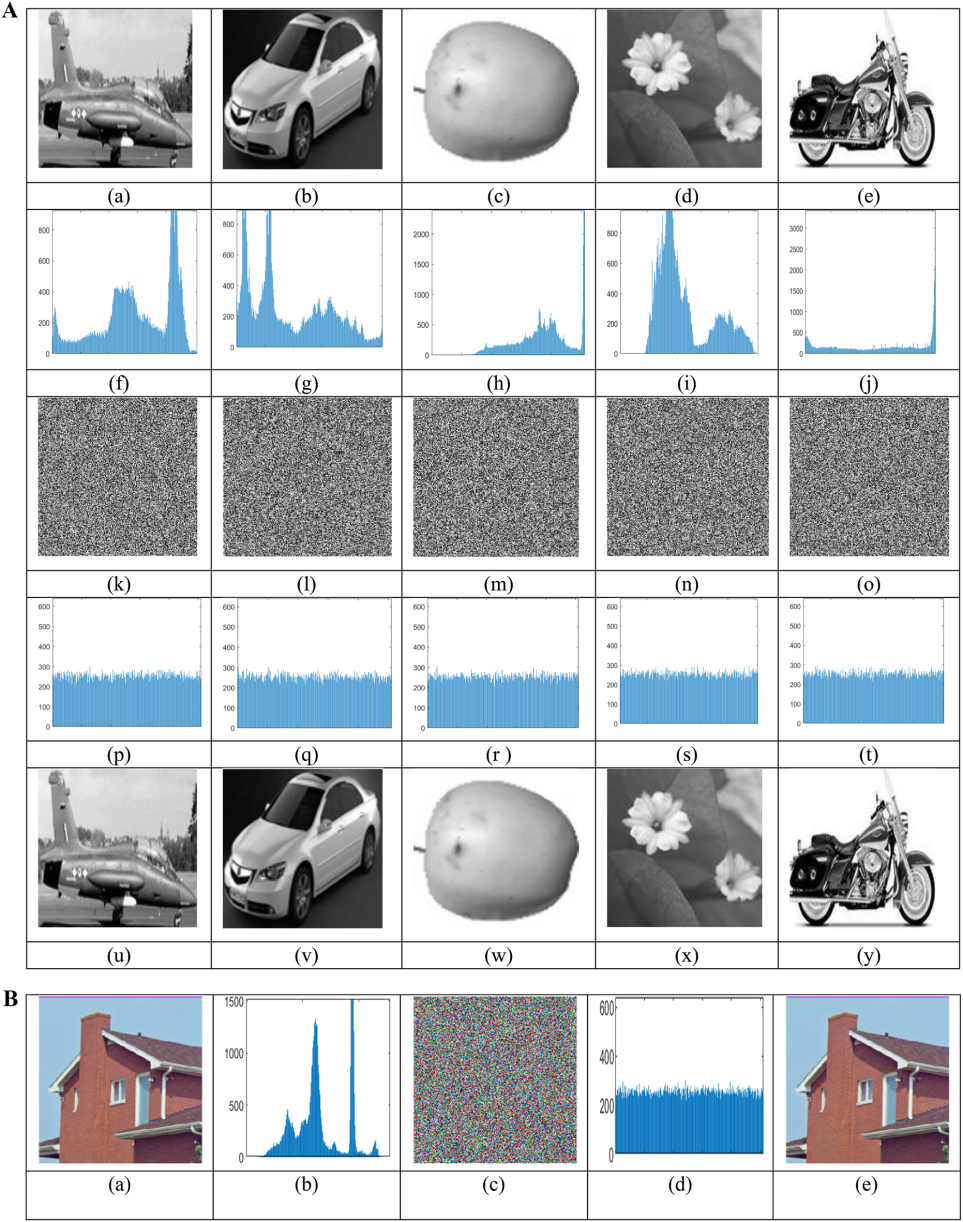
**A** Encryption analysis of proposed algorithm. (**a**–**e**) Sample input images (**f**–**j**) Histogram of sample images, (**k**–**o**) Encrypted images. (**p**–**t**) Histogram of encrypted images (**u**–**y**) Decrypted images. **B** Encryption analysis for RGB image. (**a**) RGB input image (**b**) Histogram of RGB input images, (**c**) Encrypted images. (**d**) Histogram of encrypted images (**e**) Decrypted images.

The Natural Image dataset contains 6,899 images from 8 distinct classes compiled from various sources. It can be downloaded from https://www.kaggle.com/datasets/prasunroy/natural-images. The 120 sample images were taken for the result and performance analysis. The Fig. 10A shows the encrypted and decrypted images of 5 sample images from 120 sample images and Fig. 10B displays the encrypted and decrypted image for tree colour image taken from USC-SIPI image database.

### Statistical analysis

#### Entropy analysis

A key indicator to evaluate the system’s chaos or unpredictability is entropy. It is used in information theory to measure the degree of uncertainty in predicting a random variable^30^**.** The definition of the entropy H(k) for a discrete random variable k with probability function p(k) is.

29 $$H= -\sum_{k=1}^{N}{P}_{k }{log}_{2}{P}_{k}$$

**Fig. 11:**
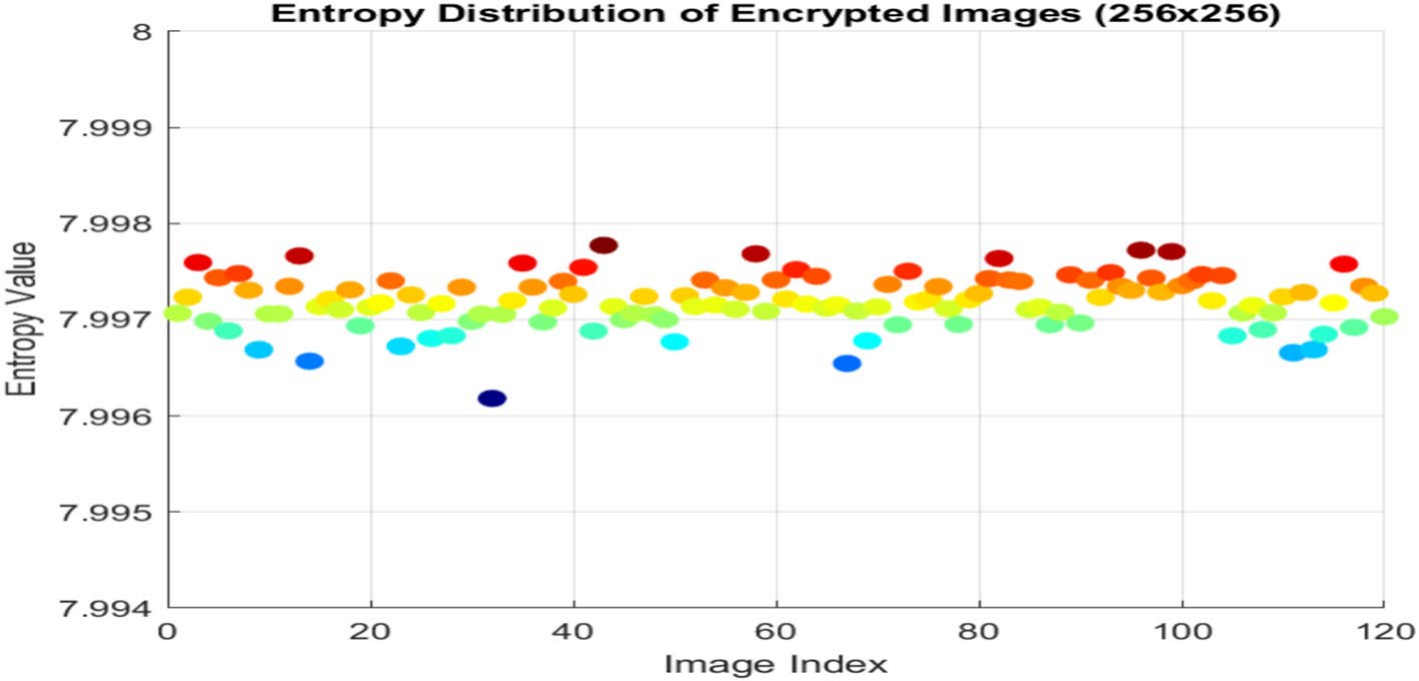
Entropy analysis of 120 sample images.

The Table 7 shows entropy values for 5 sample test images and a high degree of unpredictability for the encrypted images that are ideally near 8^31^**.** This high degree of randomness is essential to guaranteeing the security and resilience of the employed encryption technique. The entropy of the ciphered image is analysed for 120 sample images and all the ciphered image entropy values are distributed near to the critical value 8, shown in the Fig. 11. This entropy value represents that the algorithm creates more complexity to the source image which improve the efficiency of resultant cipher image.

**Table 7:** Global and local entropy analysis.

Sample images	Original image entropy	cipher image global entropy	Local entropy No. of blocks 30	Local entropy critical values		
				H^5%^left = 7.9019 H^5%^right = 7.9030	H^10%^left = 7.9017 H^10%^right = 7.9032	H^1%^left = 7.9015 H^1%^right = 7.9034
Airplane	7.4637	7.9971	7.9032	PASS	PASS	PASS
Car	7.5483	7.9971	7.9938	PASS	PASS	PASS
Fruit	6.2383	7.9973	7.9068	PASS	PASS	PASS
Flower	7.1818	7.9974	7.9071	PASS	PASS	PASS
Motorbike	6.0259	7.9974	7.9062	PASS	PASS	PASS

In order to validate the key generation process using EfficientNet-B3, Shannon entropy was calculated for each of the four seed dimensions (X_1_, Y_1_, Z_1_, and W_1_) using 256-bin histograms over the [0,1] range. It is used analysis the randomness and uniformity of the generated seed values. The resultant entropy values for initial conditions are 7.970, 7.975, 7.971, and 7.974 bits. These values ensure a high level of unpredictability and indicate statistical neutrality as they are very close to the ideal value of 8 bits for uniformly distributed image.

#### Histogram analysis

The image histogram shows the probability distribution of the image levels of intensity^32^. The histograms of the original and cypher images expected should be entirely different. The histogram of encrypted image should be flat. All intensity levels between 0 and 255 for the cypher image should be evenly spread throughout. Figure 10 displays the encrypted and decrypted 5-sample images with a histogram. As seen in Fig. 10 the histogram of the cypher images is almost uniform and evenly distributed, showing that the algorithm has excellent dispersion properties that randomise the pixels of the original image. This histogram’s uniform distribution and flatness demonstrate an unattainable adversarial statistical attack.

#### Correlation analysis

**Fig. 12:**
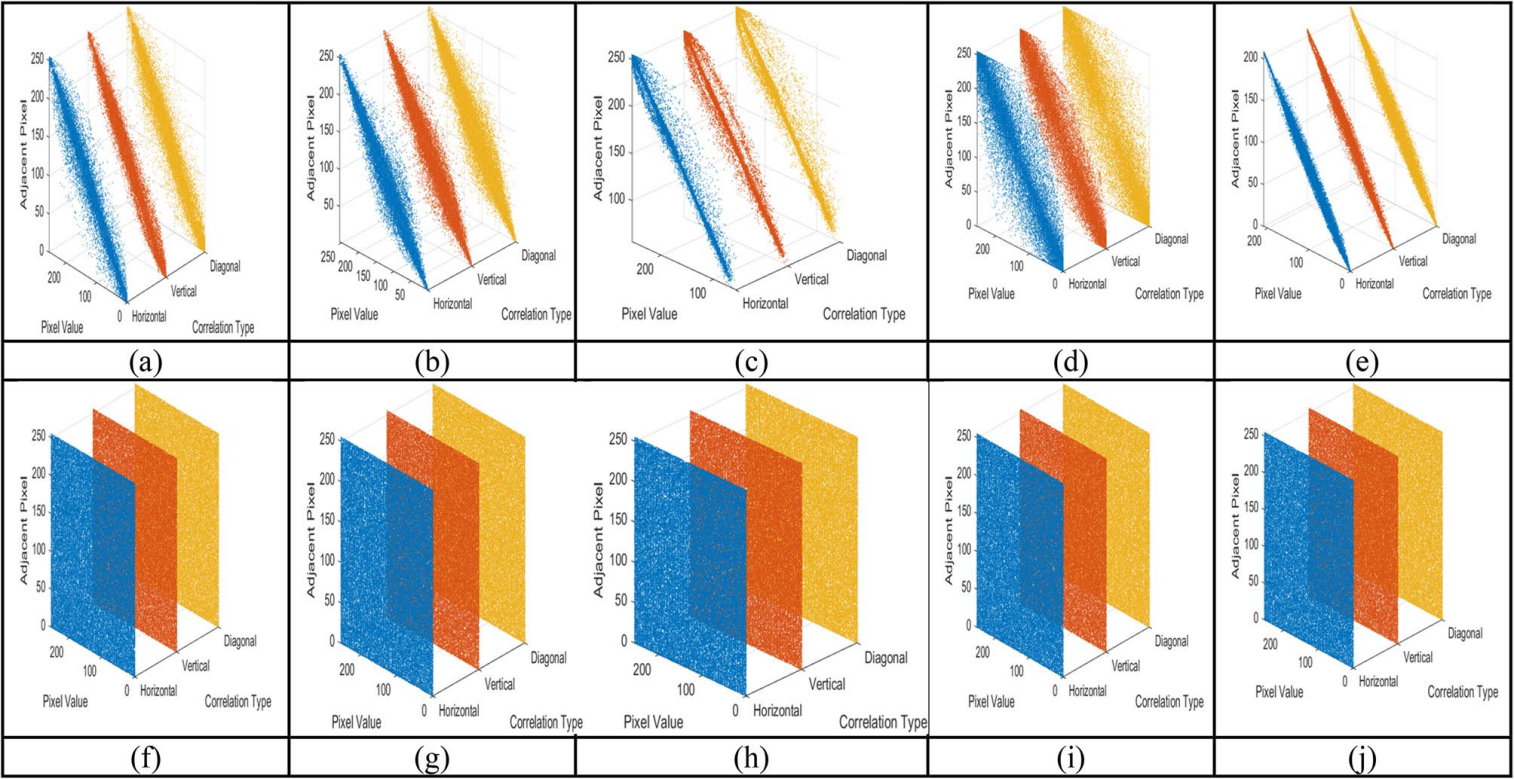
Correlation analysis. (**a**–**e**) Correlation coefficient of sample input images (**f**–**j**) Correlation coefficient of encrypted images.

The correlation coefficient analyses the relationship between the adjacent pixels in the image. The encrypted image correlation coefficient value nearer to zero indicates better encryption quality, randomizing pixel values and breaking structural patterns of the image^33^. The encryption algorithm shows its effectiveness by withstanding the statistical attack by checking the correlation coefficient. Table 8 gives the sample image’s correlation coefficients and the encrypted image’s correlation coefficient value in the horizontal, vertical, and diagonal directions. The Fig. 12 the original image correlation plot, which is linear and highly correlated in one direction, gives the visual information. The cypher image’s correlation plot is widely dispersed over its surface, giving a noisy image structure and demonstrating how the algorithm resists statistical attacks.

**Table 8:** Correlation coefficient for original and encrypted images.

Sample images	Original image correlation coefficient			Encrypted images correlation coefficient		
	Horizontal	Vertical	Diagonal	Horizontal	Vertical	Diagonal
Airplane	0.986184	0.990984	0.979561	− 0.0021456	− 0.0029691	− 0.0045902
Car	0.985660	0.987366	0.970316	0.0007193	0.0071133	0.0025958
Fruit	0.995968	0.995253	0.991229	− 0.0017832	0.0019185	0.0041977
Flower	0.995309	0.995241	0.991324	0.0009899	− 0.0019725	− 0.0011440
Motorbike	0.972005	0.981392	0.953022	0.0042312	− 0.0023287	0.0016004

30 $$corr\left(x,y\right)= \frac{cov\left(x,y\right)}{\sqrt{D(x)D(y)}}$$

Where

31 $$cov\left(x,y\right) = \frac{1}{N}\sum_{j=1}^{N}({x}_{j}-E\left(x\right))({y}_{j}-E\left(x\right))$$

32 $$E\left(x\right)= \frac{1}{N}\sum_{j=1}^{N}{x}_{j}$$

33 $$D\left(x\right)= \frac{1}{N}\sum_{j=1}^{N}{{(x}_{j}-E\left(x\right))}^{2}$$

#### Chi-squares analysis

The pictorial representation of encrypted images in the histogram plot is not sufficient to analysis the uniformity of the pixel distribution. The consistency of the encrypted pixel distribution is evaluated quantitatively using the chi-square test.

34 $${\chi }^{2}= \sum_{i=1}^{255}\frac{ {\left({O}_{i}- {E}_{i }\right)}^{2}}{{E}_{i}}$$

Where:

${\text{O}}_{\text{i}}$= Observed frequency of pixel intensities

${\text{E}}_{\text{i}}$= Expected frequency (assuming a uniform distribution)

**Fig. 13:**
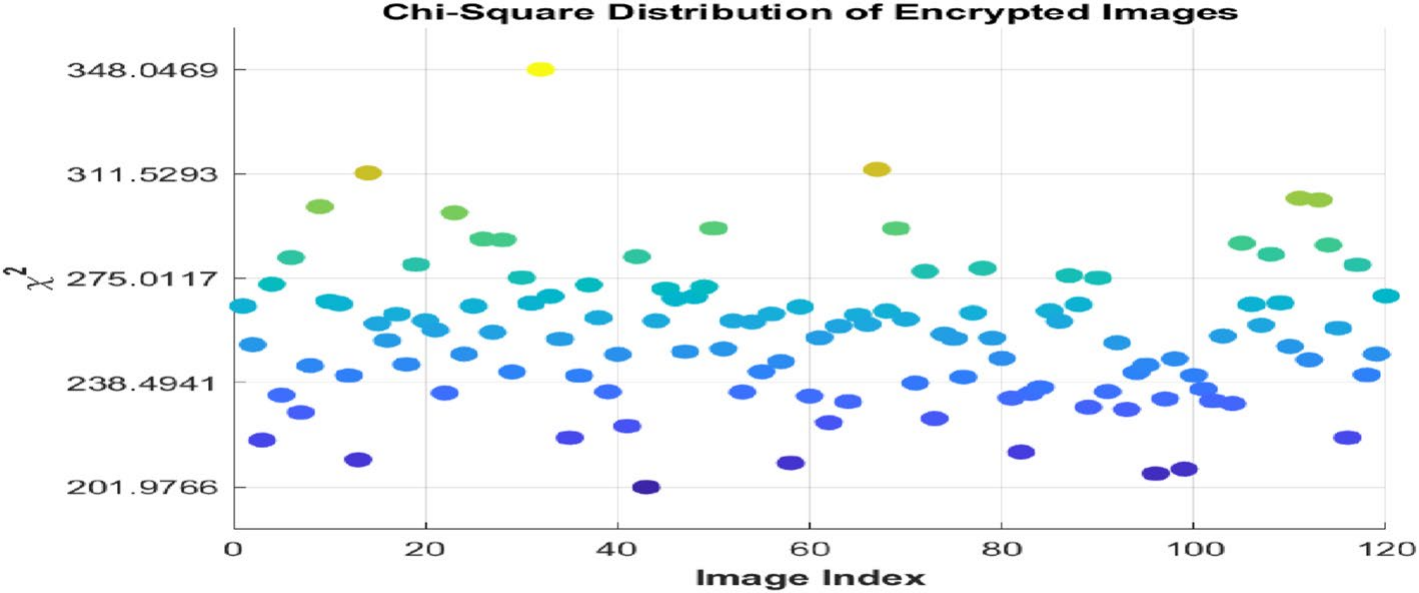
Chi square analysis for 120 sample images.

The chi square value is analysed with significance level of 5% and 1% and the P value must be higher than 0.05 to accepts the null hypothesis ^34^ which shown in the Table 9. The p-value represents the probability that the observed pixel distribution (of the encrypted image) could have occurred by random behaviour, assuming the null hypothesis is true. The chi-square value for the original image and its encrypted image for 120 sample image is plotted in the Fig. 13 Which represents all the value of cipher images lies within the critical region.

**Table 9:** chi square analysis.

Sample images	Chi-square value of original image	Chi-square value of cipher image	Critical value	
			5% = 293.2478	1% = 310.4574
Airplane	0,071,326	265.28	PASS	PASS
Car	0,060,982	262.64	PASS	PASS
Fruit	0,841,360	240.49	PASS	PASS
Flower	0,079,412	231.91	PASS	PASS
Motorbike	1,809,500	235.41	PASS	PASS

#### Deviation from ideality analysis

Deviation from the ideal value in the encrypted image is used to evaluate the divergence from the ideal encrypted histogram^35^. The equation is used to measure the DI value. For a high-performance algorithm, the histogram of the encrypted image is uniform, so the deviation from the ideality value is close to zero. The Table 10. gives the deviation from ideality for the sample image, and it shows that all the values are very near zero.

**Table 10:** Deviation from idelaity analysis.

Sample images	Deviation from ideality
Airplane	0.051605
Car	0.050323
Fruit	0.047882
Flower	0.048279
Motorbike	0.049347

35 $$DI = \sum\limits_{0}^{{255}} {\left| {\frac{{H_{{EI}} - H_{E} }}{{R \times C}}} \right|}$$

**Fig. 14:**
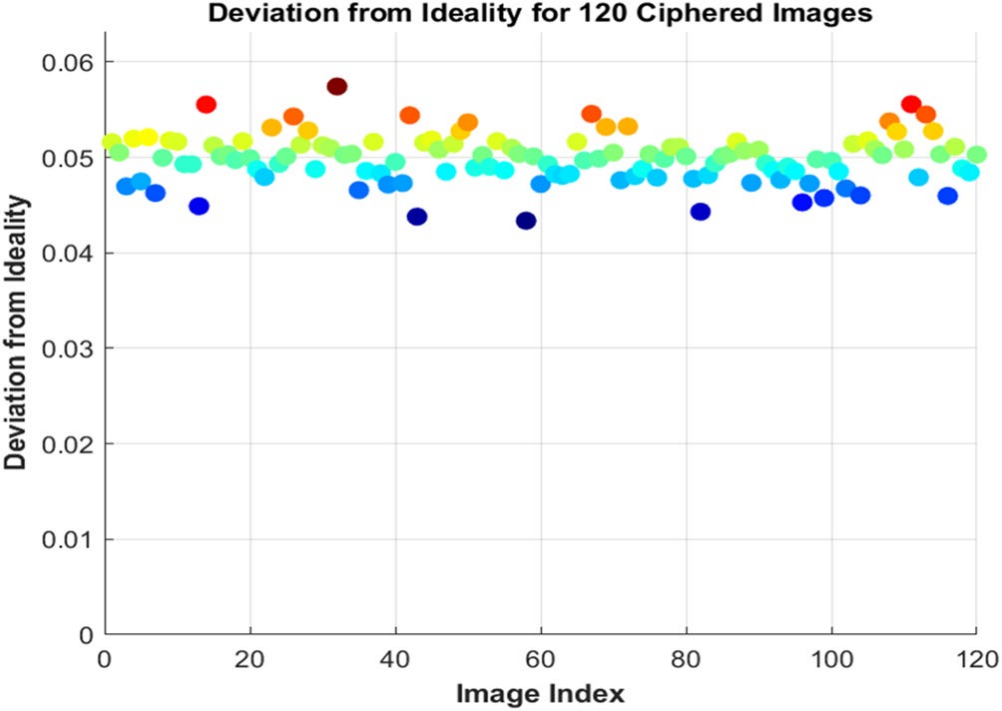
Deviation from ideality analysis.

${\text{H}}_{\text{EI}}$ Is the ideal encrypted image histogram and ${\text{H}}_{\text{E}}$ is the extracted histogram from the proposed algorithm. it is validated that the extracted histogram is almost close to the ideal histogram, there is a very small amount of divergence. The deviation from ideality for 120 sample images is plotted in Fig. 14.

### Differential attack analysis

Differential attacks aim to identify differences between two encrypted images resulting from minor variations in their plaintext counterparts. This research confirms that the encryption algorithm is resilient to a single-bit change in the plain picture^36^**.** As a result, a strong image encryption technique needs to be resistant to differential attacks. The differential attack can be evaluated by finding the Number of pixels change rate (NPCR) and Unified average change intensity (UACI) between two encrypted images. The ideal value of the NPCR is 100%, and the UACI value is 33.46%, given in the reference^37^**.** The NPCR value is calculated by finding the difference between the two cypher images, which is generated by the slight variations of the two original images. If all the pixel values are different, then it has a high NPCR value, and the proposed algorithm results in a high NPCR value near the critical value. The low UACI value derived from the algorithm ensures that the average variation in pixel intensity between two cypher images is less due to the recommended algorithm and is approximately equal to the ideal value that guarantees the privacy of the data.

36 $$NPCR = \sum_{i,j}\frac{{D}_{1}\left(i,j\right)}{R*C}$$

37 $${D }_{1}\left(i,j\right)=\left\{\begin{array}{c} 0, if {c}_{1} (i,j )= {c}_{2}(i,j) \\ 1, if {c}_{1}\left(i,j\right) \ne {c}_{2}(i,j)\end{array}\right.$$

38 $$\text{UACI}=\sum_{i,j}\frac{\left|{c}_{1} \left(i,j \right)-{c}_{2}(i,j)\right|}{255 X R*C}\times 100$$

**Fig. 15:**
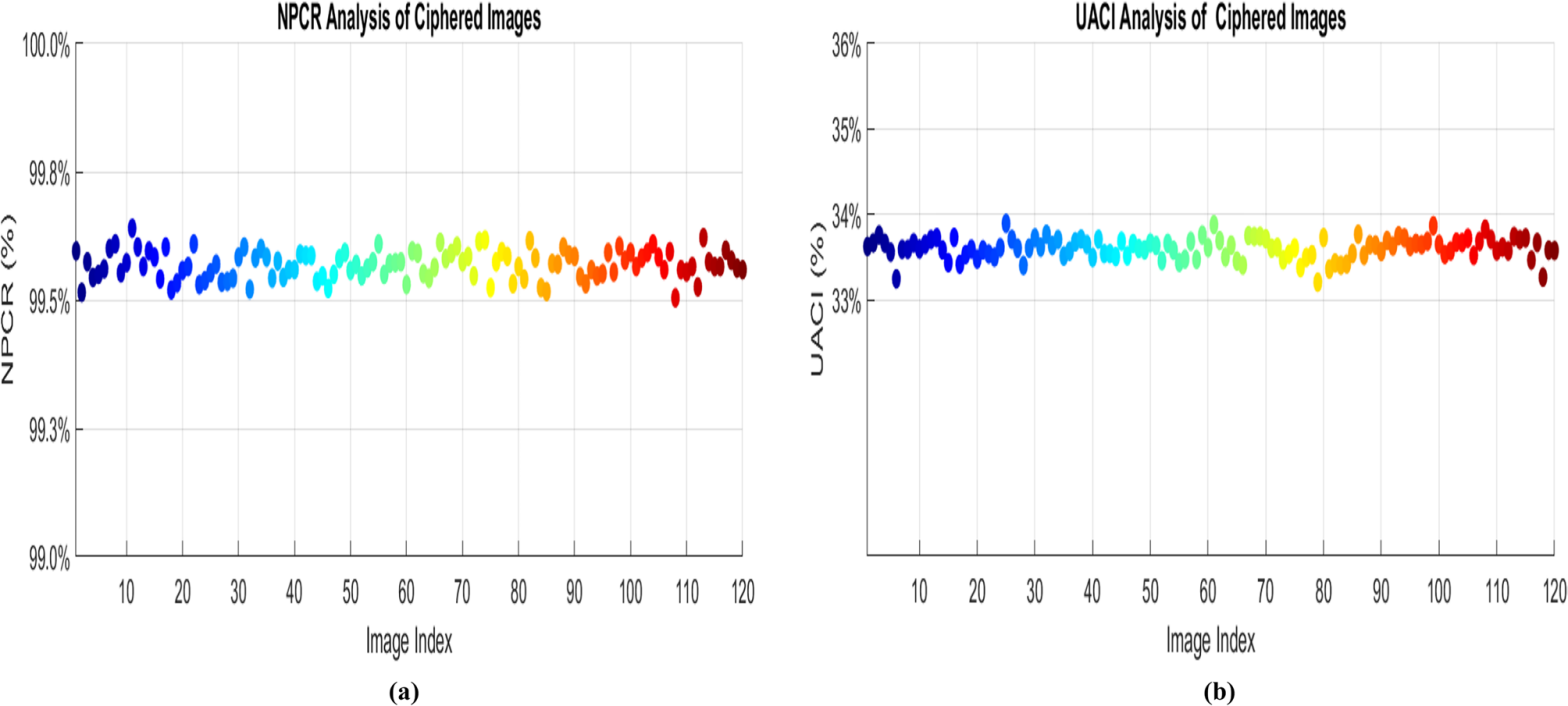
Differential attack analysis. (**a**) NPCR (**b**) UACI.

Where the two encrypted images are given as ${\text{c}}_{1} \left(\text{i},\text{j}\right)$ and ${\text{c}}_{2}(\text{i},\text{j})$ , and R and C represents the number of rows and columns of the image, respectively. Tables 11 and 12 gives the NPCR and UACI values for 5 different sample images. The NPCR and UACI values are also plotted in Fig. 15 for 120 images, showing that all the values are clustered near the critical regions, which proves the algorithm’s efficiency.

**Table 11:** NPCR analysis.

Sample images	NPCR (%)	NPCR critical values		
		N_0.05_ = 99.5693%	N_0.01_ = 99.5527%	N_0.001_ = 99.5341%
Airplane	99.96	Pass	Pass	Pass
Car	99.94	Pass	Pass	Pass
Fruit	99.99	Pass	Pass	Pass
Flower	99.97	Pass	Pass	Pass
Motorbike	99.95	Pass	Pass	Pass

**Table 12:** UACI analysis.

Sample images	UACI (%)	UACI critical values		
		U^-^_0.05_ = 33.2824% U^+^_0.05_ = 33.6447%	U^-^_0.01_ = 33.2255% U^+^_0.01_ = 33.7016%	U^-^_0.001_ = 33.1594% U^+^_0.001_ = 33.7677%
Airplane	33.42	Pass	Pass	Pass
Car	33.53	Pass	Pass	Pass
Fruit	33.42	Pass	Pass	Pass
Flower	33.46	Pass	Pass	Pass
Motorbike	33.46	Pass	Pass	Pass

### Key space analysis

The attacker has access to all encryption systems except the encryption key, which is crucial from the security perspective to protect the key^38^**.** The brute force attack is resisted if the key space is more than 2^128^ The proposed algorithm has 4 initial conditions (X_1_, Y_1_, Z_1_, W_1_) with a precision of 10^14^, 5 control parameter values, and image adaptive key generation uses SHA 256, so the total key space of the proposed encryption algorithm 2^186.^ × 2^232^ × 2^256^ = 2^(186+232+256)^ ≈ 2^674​^. Thus, the key space is enough to avoid the brute force attack.

### Key sensitivity analysis

**Fig. 16:**
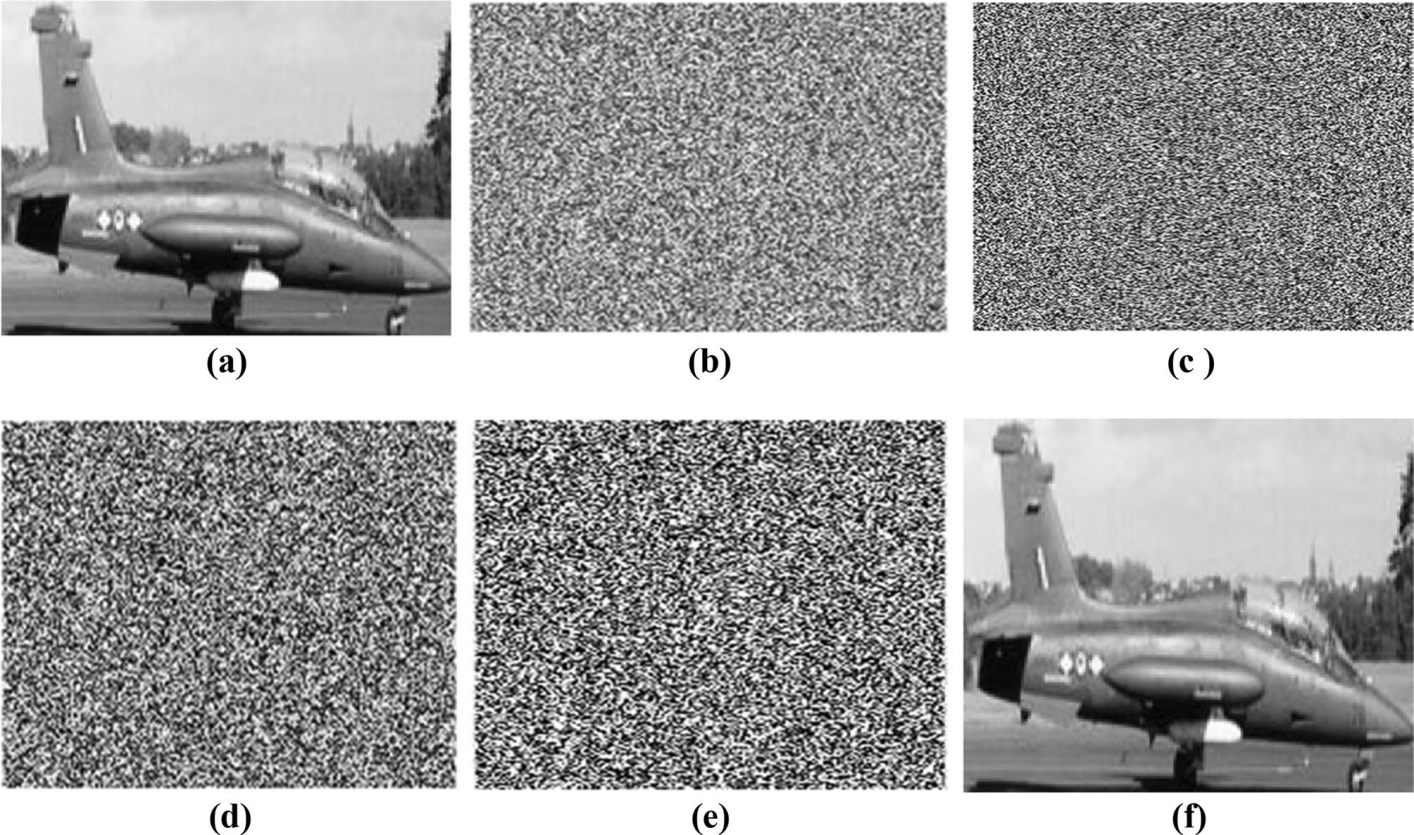
key sensitivity analysis (**a**) original data image (**b**) Decrypted image with wrong key k1 (**c**) Decrypted image with wrong key k2 (**d**) Decrypted image with wrong key k3 (**e**) Decrypted image with wrong key k4 (**f**) Decrypted image with correct key k.

The proposed technique has a high key sensitivity factor; even a slight change in the key value results in a different decryption result, which is not equal to the original image^39^. The method has a robust attack on unauthorized access using the incorrect keys given in the Table 13, and decrypting the original image is impossible. The Fig. 16 demonstrates the effect of key variation by using the wrong keys 1, 2, 3, and 4 in the algorithm, which results in the incorrect prediction of the original image and validates that the algorithm is highly sensitive to the wrong key value.

**Table 13:** Key sensitivity analysis.

Correct key (k)	Wrong key (k_1_)	Wrong key (k_2_)	Wrong key (k_3_)	Wrong key (k_4_)
X_1_ = 0.37569937711624, Y_1_ = 0.26398764600563, Z_1_ = 0.10739354407345, W_1_ = 0.44455963018465	X_2_ = 0.37677438021068, Y_2_ = 0.26427553632306, Z_2_ = 0.10748662473296, W_2_ = 0.44451915942647	X_3_ = 0.38000541171721, Y_3_ = 0.26514065471998, Z_3_ = 0.10776313827734, W_3_ = 0.44439713053851	X_4_ = 0.38216442290224, Y_4_ = 0.26571858845950, Z_4_ = 0.10794527863321, W_4_ = 0.44431525332928	X_5_ = 0.38541036978117, Y_5_ = 0.26658723608769, Z_5_ = 0.10821520855436, W_5_ = 0.44419162952140

### Error metric analysis

#### Mean squared error analysis

The two quantitative metrics used to calculate the efficiency of the proposed algorithm are MSE and PSNR. In this article, MSE is employed to calculate the mean squared difference between the original and encrypted images^40^. The higher-value MSE in Table 14. resembles strong encryption with the visually distorted structure of the original image.

39 $$MSE= \frac{1}{R*C}\sum_{i=1}^{R}\sum_{j=1}^{C}{({I}_{1}\left(i,j\right)-{C}_{1}\left(i,j\right))}^{2}$$

Where ${{\varvec{I}}}_{1}\left({\varvec{i}},{\varvec{j}}\right)$ and ${C}_{1}\left({\varvec{i}},{\varvec{j}}\right)$ are the pixel values at the position $\left({\varvec{i}},{\varvec{j}}\right)$ represents the image dimensions of the original and encrypted images. **R** represents the number of rows (height) and **C** represents the number of columns (width) of the image.

**Table 14:** MSE and PSNR analysis.

Sample images	MSE	PSNR
Airplane	10,208	8.0416
Car	11,624	7.4772
Fruit	13,197	7.9260
Flower	08,136	9.0264
Motorbike	16,104	6.0615

#### Peak signal nosie ratio

**Fig. 17:**
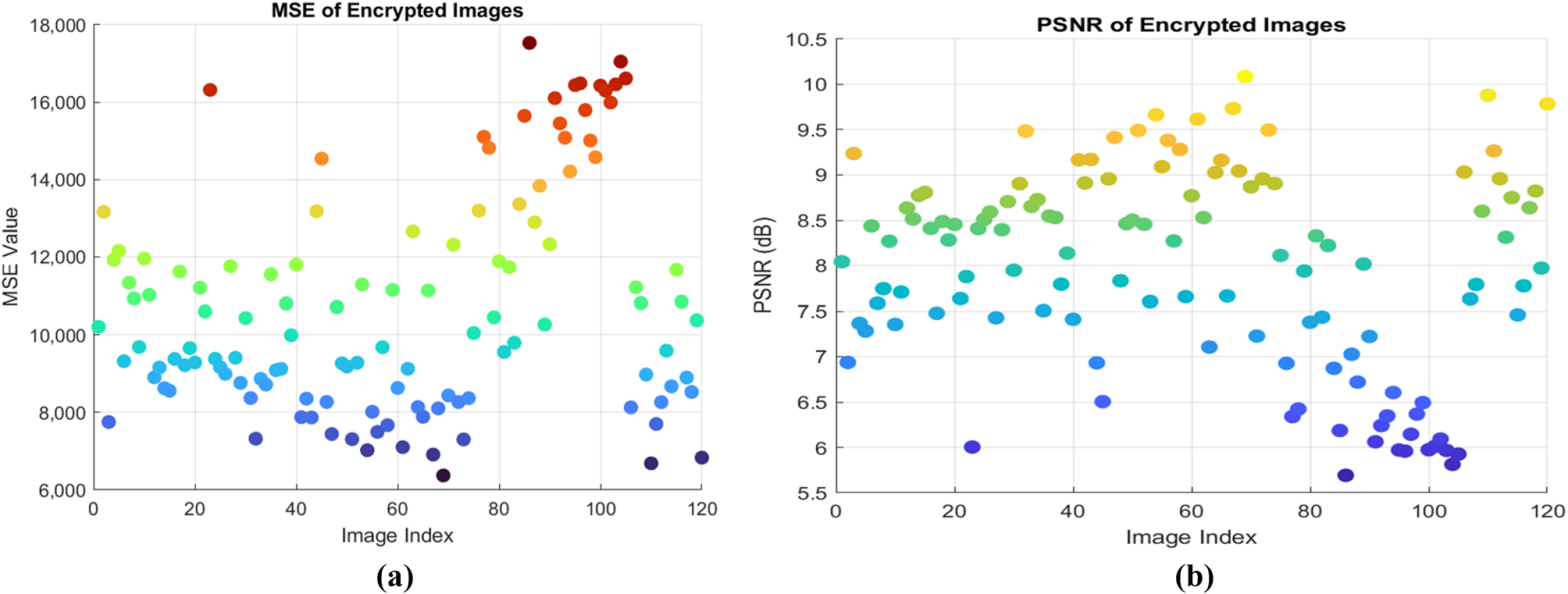
(**a**) MSE (**b**) PSNR analysis of 120 sample Images.

The peak signal-to-noise ratio (PSNR) is derived from the MSE and used to evaluate mathematically how the original image deviates from the encrypted image^41^. The low PSNR value of the encrypted image reflects the level of distortion caused by the proposed algorithm. Table 14 shows a PSNR value close to 8 infers the sound quality of image encryption. The mathematical expression of PSNR is given in Eq. 40**.** Figure 17 shows the high MSE and low PSNR values for sample 120 images.

40 $$PSNR=10{\text{log}}_{10}\frac{{255}^{2}}{MSE}$$

### Cropping attack analysis

**Fig. 18:**
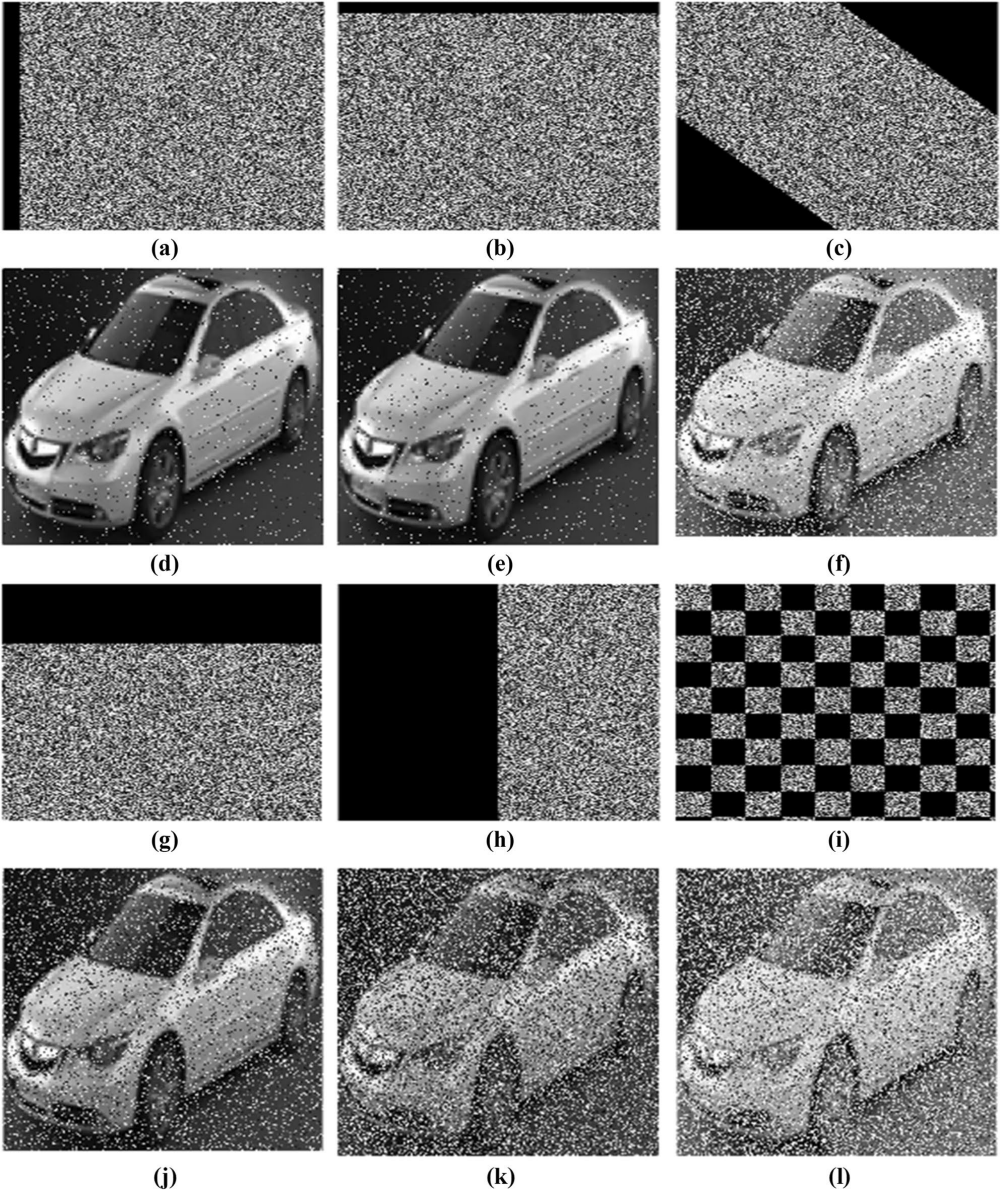
Cropping attack analysis (**a**) 5% cropped vertical cipher (**b**) 5% cropped horizontal cipher (**c**) cropped diagonal stripe (**d**) decrypted image of—‘a’ (**e**) decrypted image of—‘b’(**f**) decrypted image of–‘c’ (g) 25% horizontal cropping (**h**) 50% vertical cropping (**i**) check board cropping (**j**) decrypted image—‘g’ (**k**) decrypted image—‘h’ (**l**) decrypted image—‘i’.

The cropping attack analysis is performed to analyse the robustness of the encryption algorithm by intentionally removing some part of the encrypted data at different shapes and extracting the decrypted images after cropping. The algorithm performance is analysed using different shapes such as horizontal stripe, vertical stripe, diagonal stripe^11^, and check board cropping in the ratio of 5%, 25% and 50% shown in Fig. 18 for the sample image_002. The algorithm efficiently extracts the original image from the cropped encrypted image; very importantly, when compared to other shapes, the check board shape introduces the discontinuous cropping region, and the multi-stage encryption technique also performs partially well in this input.

### Noise attack analysis

**Fig. 19:**
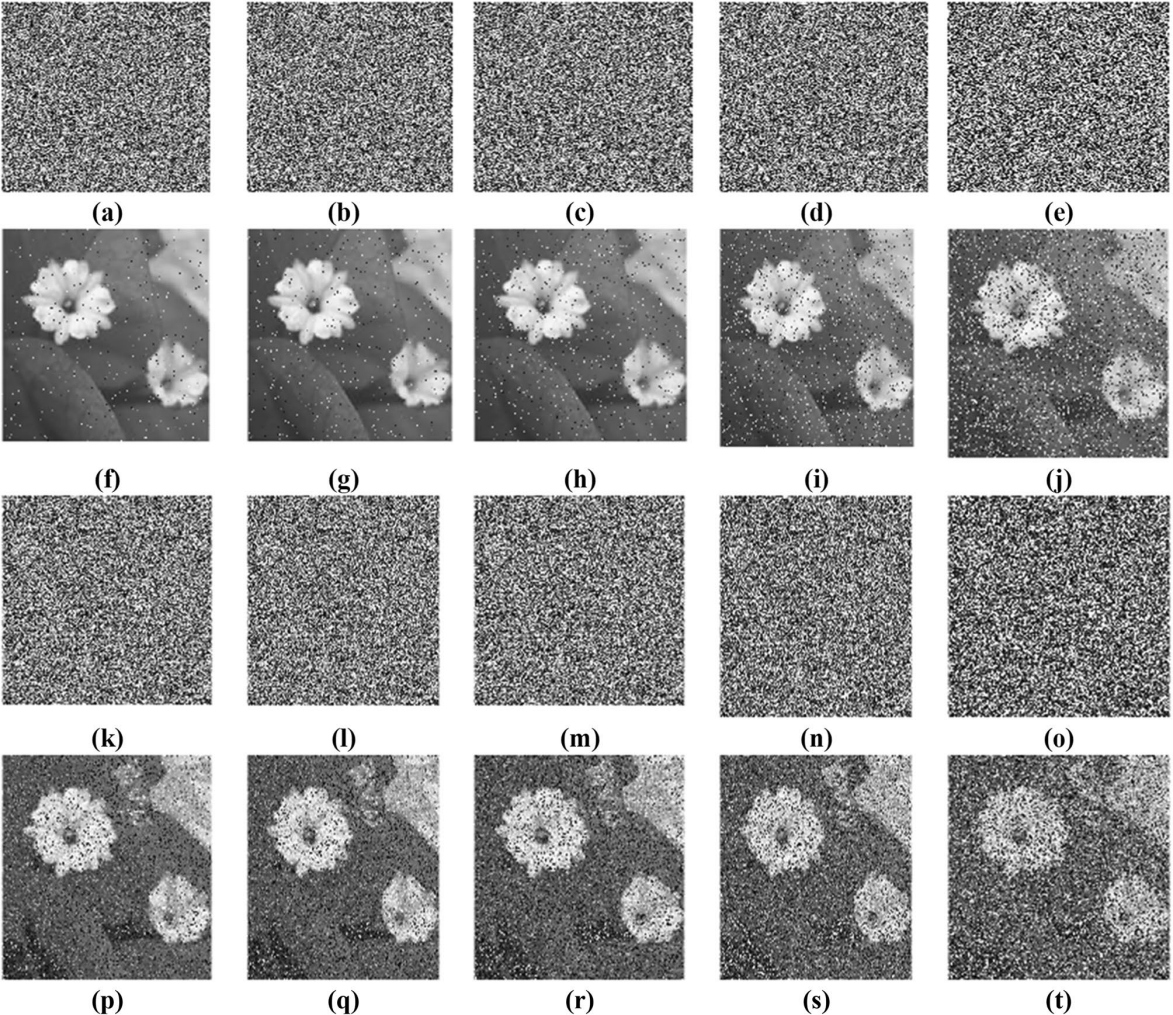
Noise Attack analysis (**a**–**e**) Encrypted image added with salt and pepper noise at the intensity level of 2%,3%,5%, 10% and 25%, (**f**–**j**) Decrypted image of salt and pepper noise added cipher image with an intensity level of 2%,3%,5%, 10% and 25%, (**k**–**o**) Encrypted image added with speckle noise at the intensity level of 2%,3%,5%, 10% and 25%, (**p**–**t**) Decrypted image of speckle noise added cipher image at the intensity level of 2%,3%,5%, 10% and 25%.

The algorithm’s competence against noise attack is performed by incorporating the salt and pepper noise and Speckle noise with varying intensity levels, such as 2%,3%, 10%, 5% and 25%^17^. The corresponding extracted images from noisy encrypted images are analysed and displayed in Fig. 19 for the sample image_003. It shows that the algorithm effectively decrypts the image and is highly robust to the noise attack.

### Chosen plain text attack and chosen ciper text attack analysis

**Fig. 20:**
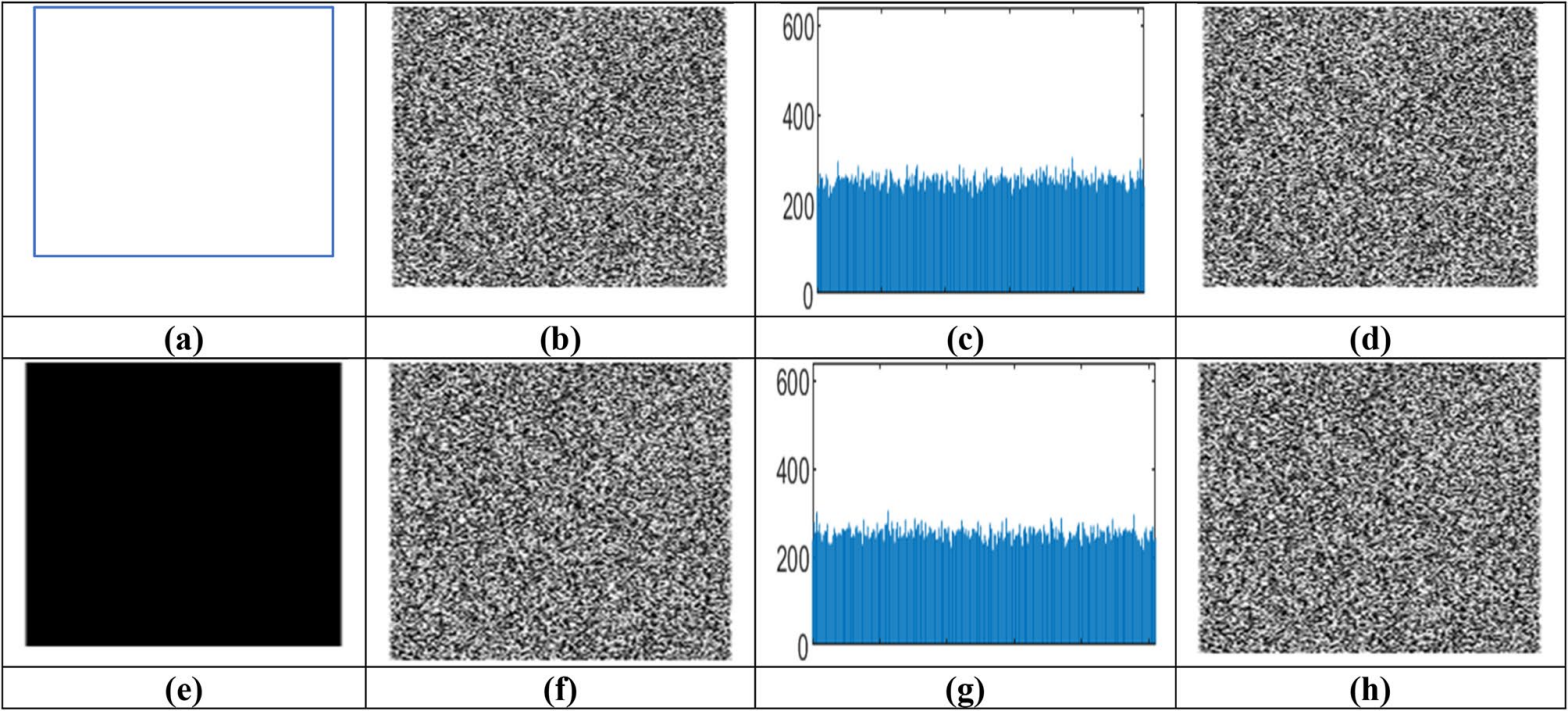
Chosen plain text and chosen cipher text attack. (**a**) All white image (**b**) Encrypted white image (**c**) Histogram of encrypted white image (**d**) Decrypted image with wrong key (**e**) All black image (**f**) Encrypted black image (**g**) Histogram of encrypted black image. (**h**) Decrypted image with wrong key for chosen cipher text.

The chosen-plaintext attack analysis is performed using an all-white image and an all-black image as input to the proposed algorithm. Figure 20 confirms that the encryption algorithm generates encrypted outputs with highly uniform histograms, effectively changing all visible patterns. Performance metrics such as entropy, NPCR, and MSE in Table 15. Indicates that the encryption process achieves values within the critical range, thereby ensuring strong resistance to chosen-plaintext attacks.

**Table 15:** Chosen plain text analysis.

Image	Original image entropy	Encrypted image entropy	NPCR	MSE
All white image	0.00	7.99	99.61	21,787.92
All black image	0.00	7.99	99.63	21,677.44

A cryptographic threat model known as a chosen ciphertext attack (CCA) occurs when an adversary deliberately chooses ciphertexts and obtains the associated decrypted plaintexts to figure out the secret key or underlying encryption logic. Each encryption instance in the recommended encryption framework is guaranteed to be extremely sensitive to input content and context, as adaptive key generation is carried out using deep semantic features obtained by Efficient Net B3. Because of its flexibility, attackers are unable to deduce consistent patterns from one decryption to another.

Different types of synthetic ciphertexts, including those generated from structured inputs such as all white and all black images, were examined to assess robustness against CCA. Key security parameters confirmed the algorithm’s robustness: entropy exceeded 7.99, NPCR was consistently above 99.60 per cent, UACI values were greater than 33.25 per cent, and MSE values showed a significant deviation from the original plaintext. The decrypted outputs showed no discernible correlation with the original inputs. These metrics show that by utilising dynamic semantic keying and efficient chaotic diffusion confusion mechanisms, the proposed strategy provides robust resilience against chosen ciphertext attacks.

### Performance comparison analysis

The proposed research compares performance parameters such as NPCR, UACI, entropy, MSE, PSNR, horizontal, vertical, and diagonal correlation coefficients, key space, and deviation from ideality with existing methodologies. Table 16 shows that our method provides excellent security performance for the Airplane_0001 and Lena images, ensuring that the proposed method is suitable for real-time open-environment applications with enhanced robustness against various types of attacks. The system shows strong resistance to differential attacks, as the NPCR values are greater than 99.6% and the UACI values remain close to the critical value of 33%. Furthermore, the correlation coefficients in the horizontal, vertical, and diagonal directions are slightly negative or near zero, thereby eliminating any inherent pixel correlation. At the same time, the entropy values are close to the ideal value of 8, confirming the high randomness of the cypher images. In addition, the key space of 2^674 is sufficiently large to resist brute-force attacks.

**Table 16:** Performance comparisons analysis.

Reference	Image	Horizontal correlation coefficient	Vertical correlation coefficient	Diagonal correlation coefficient	NPCR	UACI	Entropy	Key space
REF ^24^	Lena	− 0.0021	0.0051	0.0068	99.62	33.48	7.9990	10^62^ ≈ 2^207^
REF ^17^	Lena	− 0.0067	− 0.0176	− 0.0151	99.59	33.49	7.9970	10^42^ ≈ 2^140^
REF ^19^	Lena	− 0.0044	0.0096	− 0.0021	99.61	33.46	7.9992	10 ^77^≈ 2^256^
REF ^42^	Lena	− 0.0032	− 0.0012	− 0.0024	99.63	32.92	7.9970	10^80^ ≈ 2^265^
REF ^43^	Lena	− 0.0001	− 0.0003	− 0.0004	99.60	33.40	7.9817	10^30^ ≈ 2^449^
REF ^44^	Lena	0.0001	0.0012	− 0.0030	99.61	–	7.9976	10^105^ ≈ 2^348^
REF ^45^	Lena	0.0073	0.0115	− 0.0012	99.60	33.46	7.9897	10^111^ ≈ 2^369^
Proposed algorithm	Airplane_0001	− 0.0021	− 0.0029	− 0.0045	99.97	33.61	7.9974	10^202^ ≈ 2^674​^
Proposed algorithm	Lena	− 0.0003	− 0.0025	− 0.0012	99.94	33.35	7.9965	

### Computational complexity and runtime analysis

Encryption runtime is driven primarily by two permutation index generations, each sorting a T = 256 × 256 sequence in O (T log T). On the other hand, the formation of chaotic sequences, diffusion by XOR, and the final XOR all occur in linear time O(T). The time of the optional DNA stage, which performs rule selection and nucleotide mapping for each pixel, is likewise O(T); however, the constant factor is greater due to table lookups and encoding or decoding operations, which causes a longer elapsed time.

After extracting deep features using EfficientNet-B3, SHA-256 is used in the initial condition phase and the hashing time is insignificant when compared to the convolutional forward pass. Specifically, our CPU required approximately 0.0001 s per image to hash the 1536-dimensional feature vector, or nearly six kilobytes, while the EfficientNet-B3 forward pass dominated the mean initial-condition time of 0.1187 s. The measured times for a 256 × 256 image on our CPU-only setup were 0.1187 ± 0.0054 s for initial-condition derivation (mean and standard deviation, N = 20), 0.11814 s for the core cypher, and 0.1991s for the DNA stage. Thus the proposed method runtime results in 0.3172 s for encryption and 0.4359 s end to end when the initial condition derivation is included. Table 17 compares the runtime of the proposed encryption algorithm with existing methods and shows improved encryption speed.

**Table 17:** Run time analysis.

Algorithm	Image size	Average encryption run time(s)
Proposed algorithm	256 × 256	0.4359
Ref ^46^	256 × 256	0.4943
Ref ^47^	256 × 256	1.7590
Ref ^48^	256 × 256	0.5086
Ref ^49^	256 × 256	0.7120
Ref ^50^	256 × 256	2.1200

## Conclusion

This proposed image encryption algorithm integrates the novel 4D multi-scroll chaotic map with the EfficientNet B3 deep neural network to improve security and enhance resilience against different attacks. The image feature extracted from the EfficientNet-B3 is converted into a 256-byte hash value using SHA 256 as the initial key value to generate the chaotic sequence. The pseudo-parallel operation is executed based on the chaotic sequence. It performs multiple-stage confusion and intra/inter-pixel diffusion in the sub-blocks of the image, which gives zero-pixel correlations between adjacent pixels. Further, the key image diffusion and dynamic DNA encoding result in a high-performance encryption process and high global and local entropy, with NPCR (99.9%) and UACI (33.46%). The inter-pixel correlation in horizontal, vertical and diagonal directions is fully reduced and close to zero. The proposed algorithm enlarges the key space to **10**^**202**^**≈2**^**674​**^ , which is an important measure in the security of image encryption. This high-performance algorithm is robust against data loss, noise, and differential and statistical attacks, resulting in strong potential in real-time multimedia applications. Performance comparisons against other methodologies ensure that our algorithm has enhanced secure efficiency.

## Data Availability

The dataset was gathered from the publicly available Natural Images Dataset, which is available online. It is publicly accessible and unrestricted. P. Roy, “Natural Images,” *Kaggle Datasets*, 2019. [Online]. Available: https://www.kaggle.com/datasets/prasunroy/natural-images.
